# Trend analysis of dam inflow data using the trend accuracy index and the potential-evapotranspiration correction factor

**DOI:** 10.1038/s41598-026-40225-y

**Published:** 2026-02-20

**Authors:** Won-joon Wang, Hung Soo Kim

**Affiliations:** 1 Gyeonggi Research Institute, Suwon, South Korea; 2 Department of Civil Engineering, Inha University, Incheon, South Korea

**Keywords:** Cluster analysis, Modified Mann−Kendall test, Homogeneity test, Tank model, Trend accuracy index, Climate sciences, Environmental sciences, Hydrology, Water resources

## Abstract

Climate change intensifies rainfall and runoff variability, complicating long-term dam inflow simulation. Long-term runoff models often optimize parameters using error-based metrics such as NSE or RMSE, which reduce deviations but may fail to reproduce long-term trends. We optimize the four-tank model using the trend accuracy index (TAI) and the potential-evapotranspiration correction factor (PET-CF) to better capture inflow trends. We validate the approach by assessing trends in simulated inflows. We evaluate monthly, seasonal, and annual rainfall trends at 101 stations, examine their consistency with inflow trends, and apply K-means + + clustering. Using rainfall in the Nakdong river basin and inflow at Hapcheon Dam, we conduct clustering and trend analyses for 2000–2019 and two future periods (2021–2050, 2051–2100) under SSP2-4.5 and SSP3-7.0. Three clusters capture spatial differences in rainfall linked to station location and elevation. Across time scales, rainfall and inflow trends are not consistently aligned in either the historical or future periods. In dam inflow simulation, TAI + PET-CF achieves the highest concordance with observed inflow trends (76.4%), outperforming NSE-based calibration. Combined with rainfall trend and clustering information, the framework can support efficient water-resources management.

**Supplementary Information:**

The online version contains supplementary material available at 10.1038/s41598-026-40225-y.

## Introduction

South Korea’s rugged topography produces steep river gradients, and rainfall is concentrated during the summer rainy season, resulting in pronounced seasonal variability^1^. Efficient water-resources management is therefore essential. To secure reliable supplies for domestic and industrial use and to support hydropower generation and flood mitigation, the government has constructed hydraulic structures such as dams and barrages. However, ongoing climate change has intensified the variability of rainfall and runoff, complicating the development of management plans that must account for monthly, seasonal, and annual fluctuations^2,3^. Trend analyses of hydrological time-series data, particularly rainfall and dam inflow, can provide early insight into these dynamics, enabling central and local governments to optimize water supply and flood control strategies.

In trend analyses of hydrological time-series data (e.g., rainfall), methods such as Sen’s slope estimator and the Mann−Kendall (MK) test are commonly used. For example^4^, applied linear regression and the Kendall’s tau test to rainfall and temperature records from 66 Korea Meteorological Administration (KMA) stations. They found that, in summer, both the rainfall occurrence rate and the threshold for heavy rainfall increased, whereas in winter a significant temperature increase was observed^5^. analyzed 30 years of meteorological and hydrological time-series data for the Geumgang River basin using simple linear regression and the MK test. Their results indicated that air temperature rose in all seasons and on an annual basis, rainfall increased only in summer, and relative humidity showed a distinct decreasing trend^6^. classified flood event types based on criteria such as rainfall, snow water equivalent, air temperature, and dew point temperature, and assessed the frequency and trends of floods occurring across thousands of river basins worldwide from 1981 to 2020. Using Sen’s Slope for trend analysis, the authors found increasing trends in western North America, central Europe, and Chile, whereas a decreasing trend was observed in Australia.

Because abrupt shifts—such as sudden jumps or drops—can bias the detection of long-term tendencies, hydrological trend studies apply homogeneity tests before the trend analysis itself. Therefore, in previous hydrological trend studies, trend analysis was performed only on time-series data that passed the homogeneity test. For example^7^, performed cluster and trend analyses for 139 water-level stations across the southern and southeastern United States. After screening the data with four different homogeneity tests, they found a pronounced decreasing trend in discharge throughout the entire study period.

Cluster analysis of weather stations can also disclose rainfall patterns that are characteristic of each cluster when the grouping is based on station-specific statistics. Accordingly, rainfall trend studies at individual stations are often combined with cluster analysis. For example^8^, used a multi-objective genetic algorithm to determine both the optimal number of clusters and their interrelationships, and they applied regional frequency analysis to the rainfall data to improve the efficiency and applicability of the clustering^9^. performed trend and cluster analyses on minimum, maximum, and mean daily streamflow from 516 water-level stations across the United States for the period 1951–2009. Comparison of seasonal and annual trends among the 14 resulting clusters revealed only weak correlations between some clusters and indicated no clear association with large-scale climate indices.

In this way, station-level trend analysis improves the accuracy and reliability of analysis results through homogeneity testing and is used for efficient water-resources management in conjunction with rainfall characteristics derived from cluster analysis^10–14^. These methods are also applied to trend analysis of river discharge under future climate change scenarios. However, since future climate change scenarios do not provide data on river runoff and dam inflow, long-term runoff models such as the tank model, SWAT, and HSPF are used to estimate future simulated runoff^15–20^. To accurately estimate future simulated runoff, parameter optimization of the long-term runoff model is performed using past hydrological time-series data^21–24^. In this process, past actual runoff is set as the reference value, and the parameters are optimized to minimize the difference between the simulated and actual values using fitness functions such as the Nash−Sutcliffe efficiency (NSE) and root-mean-square error (RMSE). However, the previously used objective functions, such as NSE and RMSE, focus on minimizing deviation and thus have limitations in reproducing the statistical characteristics of long-term hydrological time-series data, particularly trend patterns. Therefore, to obtain accurate results in trend analysis of runoff under future climate change scenarios, it is necessary to develop a new objective function that can properly capture trends in actual runoff and validate its performance through verification.

This study applied homogeneity tests and the modified Mann−Kendall (MK) test to rainfall data from 101 weather stations in the Nakdong River basin and to inflow records for Hapcheon Dam, evaluating monthly, seasonal, and annual trends for a past period (2000–2019) and two future periods (2021–2050 and 2051–2100). In addition, using the tank model, we performed trend analysis of simulated dam inflow for each future scenario (SSP2-4.5 and SSP3-7.0) and optimized 13 parameters: 12 tank parameters (excluding the initial storage of each tank) and the Potential-Evapotranspiration Correction Factor (PET-CF). Furthermore, during optimization, the newly developed Trend Accuracy Index was set as the objective function, and its performance was compared across cases when applied alongside the PET-CF. Therefore, we selected the case with the highest trend concordance rate relative to observed dam inflow for trend analysis of simulated dam inflow under future climate change scenarios. Finally, we linked the rainfall and dam inflow trend results to cluster analysis results based on station rainfall characteristics and altitude, so that they could be used for efficient water-resources management.

## Materials and methods

**Fig. 1:**
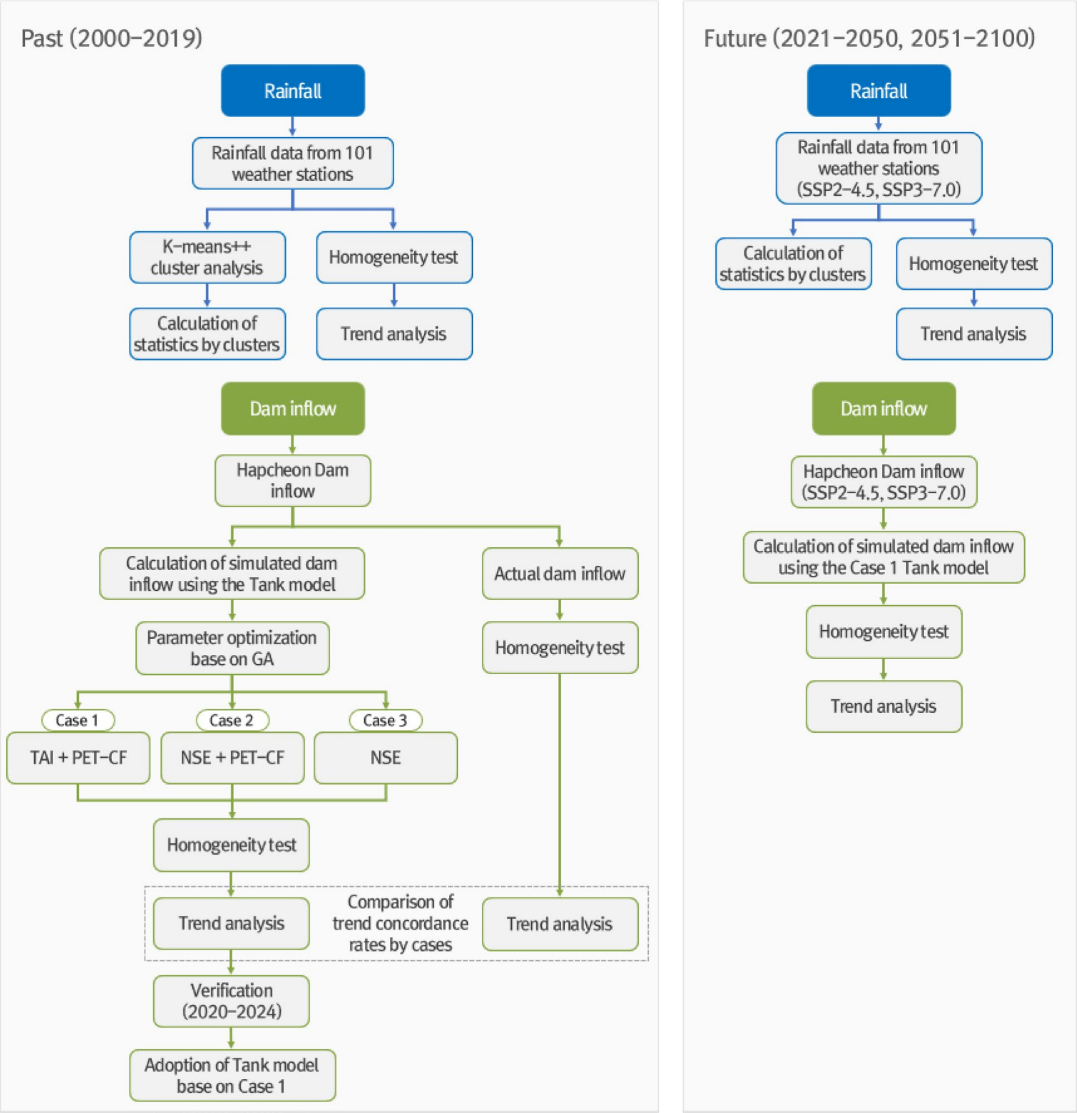
Flowchart for trend and cluster analyses of rainfall and dam inflow.

Figure 1 shows the flowchart for the trend and cluster analyses of rainfall data from 101 weather stations in the Nakdong River basin and dam inflow data for Hapcheon Dam.

### Hydrometeorological characteristics of the Nakdong river basin

**Fig. 2:**
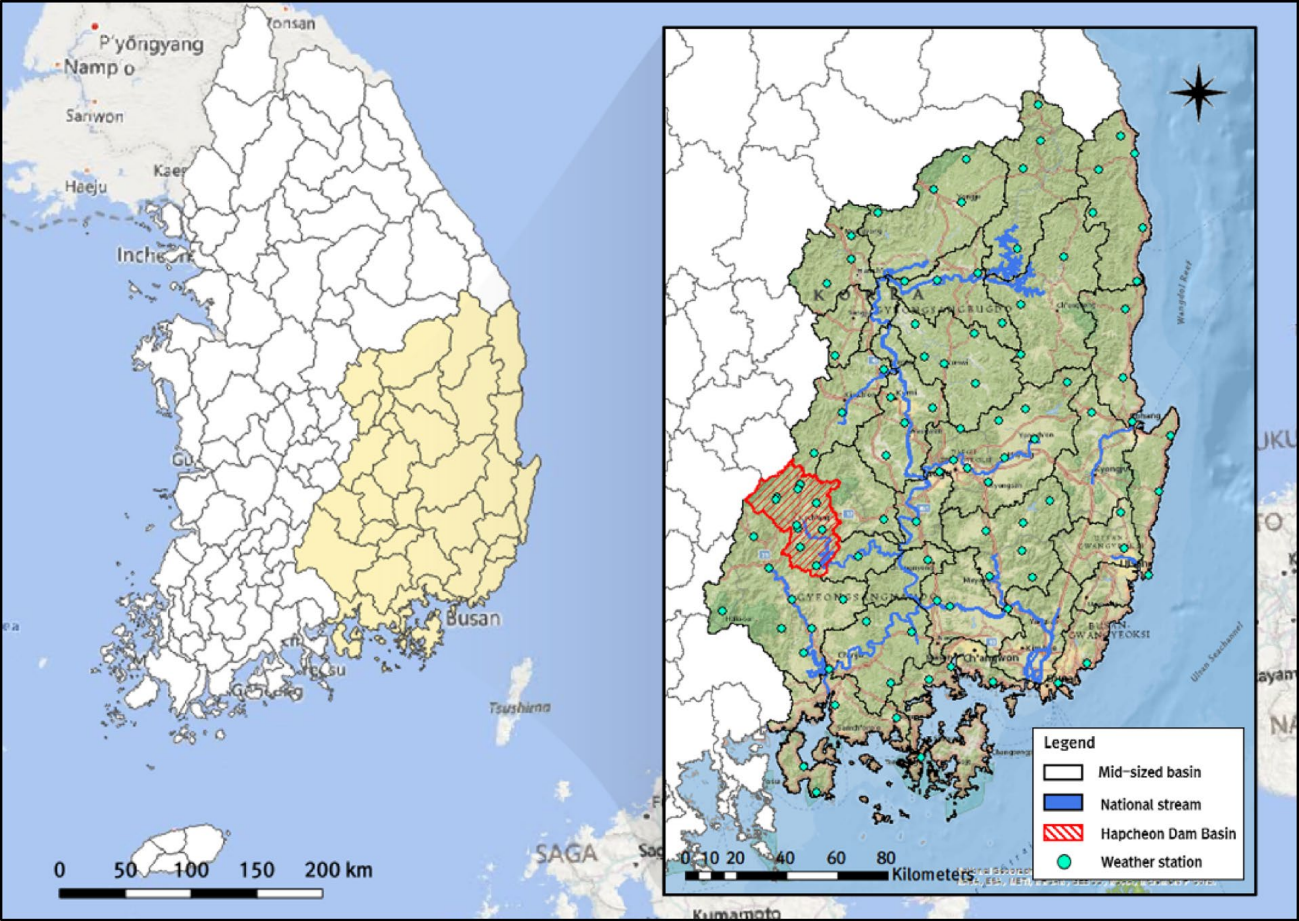
Hydrological and geomorphological characteristics of the Nakdong River basin and the distribution of weather stations in Korea. The maps were created using ArcGIS Pro 3.6.1 (https://www.esri.com/en-us/arcgis/products/arcgis-pro/overview) with the National Geographic World Map basemap and QGIS 3.10.14 (https://qgis.org) with a Bing Maps road basemap (Microsoft).

The Nakdong River basin consists of 33 mid-sized basins and covers a total area of 31,785 km², making it the second-largest of Korea’s four major river basins^25^. Geomorphologically, the Taebaek and Sobaek Mountains lie within the basin, causing the national river’s main stem to meander in a four-stage detour before discharging into the South Sea. Compared with other national rivers, the Nakdong River has a very gentle gradient, a wide channel, and severe erosion, and it has experienced frequent flood damage in the past. Despite the construction of dams and weirs intended to mitigate flooding, the basin continues to experience typhoon-induced floods, most recently during Typhoons Chaba (2016), Kong-rey (2018), and Mitag (2019)^26–28^. Consequently, the government is establishing not only structural measures for the Nakdong River basin but also non-structural measures such as the Long-Term Water Resources Comprehensive Plan. Figure 2 shows the locations of 101 weather stations used in the cluster and trend analyses of rainfall in the Nakdong River basin.

In addition to analyzing rainfall, this study selected the Hapcheon Dam basin—a mid-size basin in the southwestern portion of the Nakdong River basin—as a pilot area for examining trends in dam inflow. The Hapcheon Dam basin is located upstream of the Hwang River, a tributary of the Nakdong River, and is unaffected by artificial discharge regulation implemented at the upstream Andong and Imha dams; it is therefore well suited to trend analysis of dam inflow. The basin spans approximately 928 km², more than 70% of which is forested. Its soils are dominated by granite and gneiss, conferring high permeability. Hapcheon Dam, a multipurpose dam located at the basin outlet, was completed in 1988 and provides flood control, hydropower, and water supply.

**Fig. 3:**
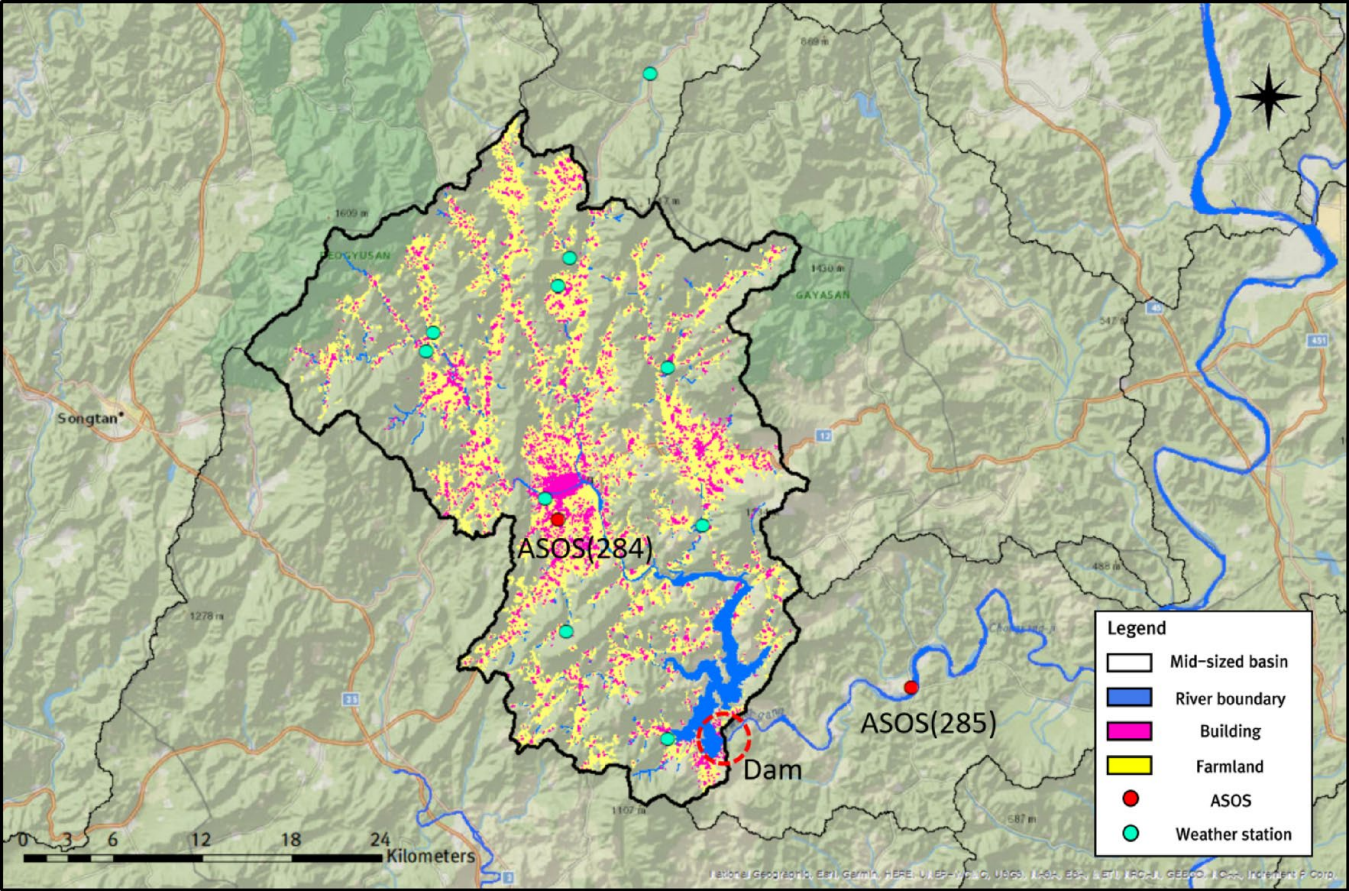
Hydrological and geomorphological characteristics of the Hapcheon Dam Basin and the distribution of 12 weather stations. The map was created using ArcGIS Pro 3.6.1 (https://www.esri.com/en-us/arcgis/products/arcgis-pro/overview) with the National Geographic World Map basemap.

This study collected and used multiple datasets for trend analysis, cluster analysis, and runoff simulation using the tank model. In addition to historical rainfall data (2000–2019), mean, minimum, and maximum air temperature data—required to estimate potential evapotranspiration—were obtained from the Korea Meteorological Administration (KMA) Open MET Data Portal. Furthermore, observed Hapcheon Dam inflow data used for historical trend analysis were obtained from K-water’s Water Information Portal. Finally, rainfall, temperature, and other scenario variables for SSP2-4.5 and SSP3-7.0 for 2021–2050 and 2051–2100 were obtained from the Korea Meteorological Administration Climate Information Portal. All datasets used in this study are publicly accessible and freely available. Figure 3 illustrates the basin’s hydrological and geomorphological characteristics and the locations of 12 weather stations, including two automated synoptic observing system (ASOS) stations.

### K-means + + algorithm

Cluster analysis groups objects with similar attributes^29^. In this study, we applied the non-hierarchical K-means + + algorithm to 101 weather stations in the Nakdong River basin^30–32^. The traditional K-means algorithm randomly assigns initial centroids, which can yield poor clustering when the chosen centroids are too close. By contrast, the K-means + + algorithm randomly selects the first centroid, calculates the distances from all remaining points to this centroid, and chooses the next centroid with a probability proportional to the squared distance. This process is repeated to sequentially place subsequent centroids, resulting in clusters in which the distances between centroids are maximized compared with the standard K-means algorithm. The algorithm then iterates to minimize the sum of squared Euclidean distances between each point and its cluster centroid.

However, to obtain reliable clustering results with the K-means + + algorithm, the optimal number of clusters, k, must be determined beforehand. In this study, the Within-Cluster Sum of Squares (WSS), F-test, and the Silhouette method were used to determine the optimal number of clusters.

### Homogeneity test

Homogeneity testing detects abrupt changes in time-series data that can distort population characteristics (e.g., trends), as shown in Eq. (1). Reliable rainfall trend analysis therefore requires independent, homogeneous, high-quality data. Any series that fails a homogeneity test can mislead trend assessment, so data quality must be verified with such tests before analysis^33–37^.

1 $$\:{x}_{i}=\left\{\begin{array}{c}\mu\:+{\epsilon}_{i}\\\:\mu\:+\varDelta\:+{\epsilon}_{i}\end{array}\right.\:\:\:\:\:\:\:\:\:\:\begin{array}{l}\mathrm{i}=1,\:2,\:\cdots\:,\:\mathrm{m}\\\:\mathrm{i}=\mathrm{m}+1,\:\mathrm{m}+2,\:\cdots\:,\:\mathrm{n}\end{array}$$

$\:{x}_{i}$: *The i-th observation value in the time-series*.

*m: Change-point position*.

$\:\mu\:$: *Population mean before the change-point*.

$\:\varDelta\:$: *Mean shift occurring after the change-point*.

$\:{\epsilon}_{i}$: *Random error term*.

In this study, the Standard Normal Homogeneity Test (SNHT), Pettitt’s Test, and Buishand Range Test (BRT) were applied to rainfall and dam inflow data. Trend analysis was performed only on time-series data that passed all three homogeneity tests.

### Modified Mann-Kendall test

The MK test is a widely used nonparametric hypothesis test for identifying trends in time-series data^38–42^. In the MK test, the null hypothesis assumes that there is no trend in the time-series data and that the data are statistically independent, while the alternative hypothesis assumes that there is an increasing or decreasing trend in the time-series data. When the time-series data are denoted as $\:{X}_{i}$, $\:{X}_{i+1}$, …, $\:{X}_{n}$, rejection of the null hypothesis based on the test statistic S and its variance indicates that a trend exists in the data. In this study, the significance level was set to 0.05. The null hypothesis was rejected when *p* < 0.05 and the test statistic S satisfied the conditions in Eq. (2), indicating that a trend exists in the time-series data. Additionally, to assess the direction of the trend (increasing, decreasing) and the magnitude of variability in the time-series data, the statistical measure Z is used as the criterion, as shown in Eq. (3).

2 $$\:\left|S\right|>{z}_{1-2/\alpha\:}\sqrt{V\left(S\right)}$$

3 $$\:\mathrm{Z}=\left\{\begin{array}{c}\frac{S-1}{\sqrt{Var\left(S\right)}}\\\:0\\\:\frac{S+1}{\sqrt{Var\left(S\right)}}\end{array}\:\:\:\:\:\:\:\:\:\:\begin{array}{c}S>0\\\:S=0\\\:S<0\end{array}\right.$$

However, the MK test risks misclassifying non-trending data as trending when the time-series data used in the analysis have autocorrelation. Therefore, in this study, the modified MK test was used to analyze the trend in rainfall data and dam inflow data^43–47^. The modified MK test is a method that uses a modified variance $\:{V}^{\mathrm{*}}\left(S\right)$ by applying a correction coefficient $\:n/{n}^{\mathrm{*}}$ to $\:V\left(S\right)$ to remove autocorrelation present in the time-series data. Equation (4) shows the formula for calculating $\:{V}^{\mathrm{*}}\left(S\right)$ with the correction coefficient, and Eq. (5) shows the formula for calculating $\:{n}^{\mathrm{*}}$, the effective sample size used when performing the modified MK test with lag-1 ($\:{\rho\:}_{1}$ is the correlation coefficient of the lag-1 series).

4 $$\:{V}^{*}\left(\mathrm{S}\right)=V\left(S\right)\frac{n}{{n}^{*}}$$

5 $$\:{n}^{*}=\:\frac{n}{1+2\frac{{\rho\:}_{1}^{n+1}-n{\rho\:}_{1}^{2}+(n-1){\rho\:}_{1}}{n{({\rho\:}_{1}-1)}^{2}}}$$

### Dam inflow estimation using a four-tank model

**Fig. 4:**
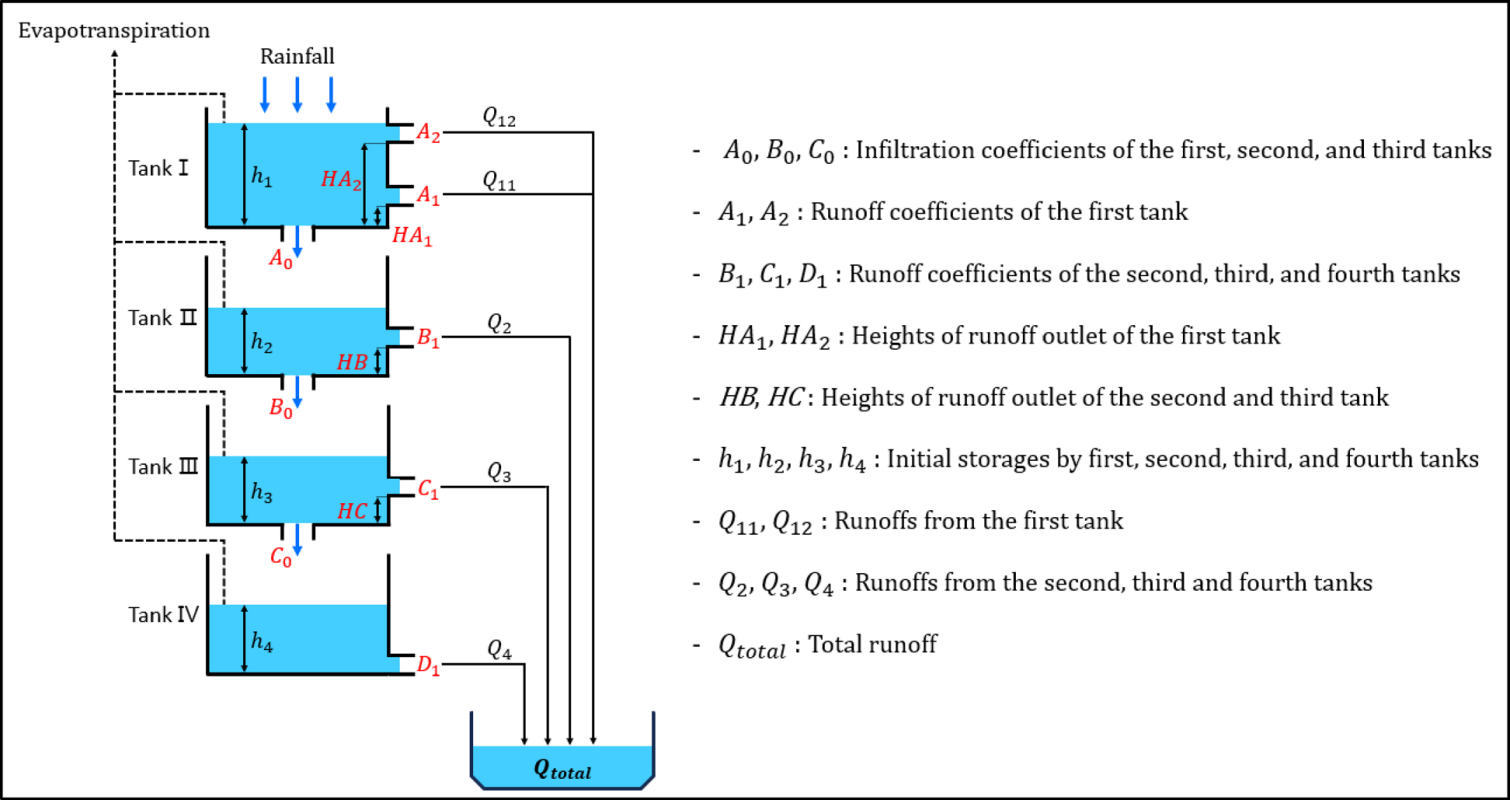
Schematic diagram of the tank model.

In this study, we used a Four-Tank Model (Fig. 4) to simulate future dam inflow and analyze trends in Hapcheon Dam inflow under future climate change scenarios. The tank model requires fewer data and fewer computational resources than other long-term runoff models such as SWAT and PRMS, while still providing highly accurate simulated runoff. Because the long-term simulation was performed at a monthly time step rather than at hourly or daily time steps, rainfall and evapotranspiration (inputs) and dam inflow (output) were aggregated to monthly values and used in the tank model. Therefore, considering these characteristics, the initial storage of each tank in the Four-Tank Model was set to zero, and rainfall was reflected in the storage of each tank without delay. Additionally, evapotranspiration was applied by sequentially subtracting it from the storage of the first tank; if evapotranspiration exceeded the storage of the first tank, it was sequentially subtracted from the storage of the second, third, and fourth tanks.

Finally, we used a genetic algorithm to optimize 13 parameters: PET-CF and 12 Four-Tank Model parameters, excluding the four initial storage values of the tanks. Equation (6) gives the total runoff computed using the Four-Tank Model. Because runoff computed using the tank model is expressed in the same units ($\:mm/day$) as rainfall, Eq. (6) was used to convert it to the units ($\:{m}^{3}/sec$) used for observed dam inflow.

6 $$\:{Q}_{total}=({Q}_{11}+{Q}_{12}+{Q}_{2}+{Q}_{3}+{Q}_{4})\times\:\frac{Area}{86.4}$$

$$\:{Q}_{total}:\:Total\:runoff\:({m}^{3}/sec$$ *)*

$\:{Q}_{11}$, $\:{Q}_{12}$: *Runoffs from the first tank (*$\:mm/day$*)*.

$\:{Q}_{2}$, $\:{Q}_{3}$, $\:{Q}_{4}$: *Runoffs from the second*,* third and fourth tanks (*$\:mm/day$*)*.

*Area: Basin area (*$\:{km}^{2}$*)*.

#### Application of potential-evapotranspiration correction factor

To calculate the inflow of the Hapcheon Dam using a Four-Tank Model, data from 12 weather stations (ASOS, AWS, Ministry of Environment, Korea Water Resources Corporation) distributed around the Hapcheon Dam basin were used, as shown in Fig. 3. Among these, rainfall data were converted to areal rainfall using the Thiessen polygon method based on point measurements from the 12 weather stations. Potential-Evapotranspiration was calculated at two ASOS stations (284 and 285), for which variables such as rainfall and temperature are systematically managed, and then converted to areal values using the Thiessen polygon method. Although the FAO Penman−Monteith method is commonly used to calculate potential evapotranspiration, the KMA SSP climate scenario dataset used in this study does not include the solar radiation data required for the FAO Penman−Monteith method. Therefore, in this study, the Hargreaves-Samani method was used to estimate potential evapotranspiration based on temperature and extraterrestrial radiation data^48–53^. Although this method is less accurate than the FAO Penman-Monteith method, it has the advantages of being less complex and more suitable for regions with limited data availability. Equation (7) gives the Hargreaves−Samani equation for potential evapotranspiration, and Eq. (8) gives the equation for extraterrestrial radiation.

7 $$\:{ET}_{O}=({K}_{ET}\cdot\:{R}_{a}\cdot\:({T}_{mean}+17.8)\cdot\:\sqrt{{T}_{max}-{T}_{min}}$$

$$\:{ET}_{O}:\:Potential\:evapotranspiration\:(mm/day$$ *)*

$$\:{K}_{ET}$$: *Correction factor*.

$$\:{R}_{a}:\:Extraterrestrial\:radiation\:(MJ/{m}^{2}/day$$ *)*

$\:{T}_{mean}$: *Monthly average temperature (*°C*)*.

$\:{T}_{max}$: *Monthly maximum temperature (*°C*)*.

$\:{T}_{min}$: *Monthly minimum temperature (*°C*)*.

8 $$\:{R}_{a}=\frac{24\cdot\:60}{\pi\:}\cdot\:Gsc\cdot\:dr\cdot\:\left[{\omega\:}_{s}\cdot\:\mathrm{sin}\left(\varnothing\:\right)\cdot\:\mathrm{sin}\left(\delta\:\right)+\mathrm{cos}\left(\varnothing\:\right)\cdot\:\mathrm{cos}\left(\delta\:\right)\cdot\:\mathrm{sin}\left({\omega\:}_{s}\right)\right]$$

$$\:Gsc=0.0820:\:Solar\:constant\:(MJ/{m}^{2}/min$$ *)*

$\:\varnothing\:$: *Geographic latitude (rad)*.

$\:\delta\:$: *Solar declination (rad)*.

$\:{\omega\:}_{s}$: *Sunset hour angle (rad)*.

$\:dr$: *Inverse relative distance Earth − Sun*.

The correction factor $\:{K}_{ET}$ used in the Hargreaves-Samani method’s potential evapotranspiration calculation formula is generally set to 0.0023, derived from an analysis of eight years of meteorological data in California, USA. However, this correction factor has been found to be unsuitable for the climatic characteristics of the Nakdong River basin in Korea. Therefore, in this study, the correction factors for each ASOS observation station proposed by^54^ were used. Using these correction factors yields results similar to those obtained using the FAO Penman−Monteith method. Therefore, in this study, the correction factor $\:{K}_{ET}$ was set to 0.00148 for weather station 284 and 0.00150 for weather station 285.

However, if potential evapotranspiration estimated using the Hargreaves−Samani method is applied directly to the tank model without adjustment, tank storage may be excessively reduced, potentially leading to underestimation of simulated dam inflow. Therefore, in this study, the modified evapotranspiration $\:{ET}_{modi}$, obtained by applying the correction factor $\:{\alpha\:}_{modi}$ to the potential evapotranspiration as shown in Eq. (9), was used in the tank model.

9 $$\:{ET}_{modi}={\alpha\:}_{modi}\cdot\:{ET}_{O}$$

#### Calculation of optimal parameters for a tank model using a genetic algorithm

A genetic algorithm is a global optimization method inspired by the evolutionary processes of living organisms and is widely used in hydrology to optimize parameters of short- and long-term rainfall−runoff models. In this study, we used a genetic algorithm to optimize 13 parameters (12 Four-Tank Model parameters and PET-CF) to estimate Hapcheon Dam inflow under SSP climate change scenarios. The search range for the 13 parameters optimized by the genetic algorithm was set as shown in Eq. (10).

10 $$\:0\le\:{A}_{0},{B}_{0},\:{C}_{0},{A}_{1},{A}_{2},{B}_{1},{C}_{1},{D}_{1}\le\:2.0460\le\:{HA}_{1},{HA}_{2},HB,HC\le\:\mathrm{2,046}\:0\le\:{\alpha\:}_{modi}\le\:1.023$$

To improve trend concordance between simulated and observed dam inflow, we used the newly developed TAI as the fitness function. Additionally, parent chromosomes were selected based on fitness using both rank selection and an elitism-preserving selection method. In the case of the rank selection method, the fitness of chromosomes was ranked in descending order based on the TAI, as shown in Eq. (11), and then the selection probability for each chromosome was calculated and applied.

11 $$\:Selection\:probability=\frac{\frac{1}{RANK\left(Chromosome\right)}}{\sum\:\left(\frac{1}{RANK\left(Chromosome\right)}\right)}$$

**Fig. 5:**
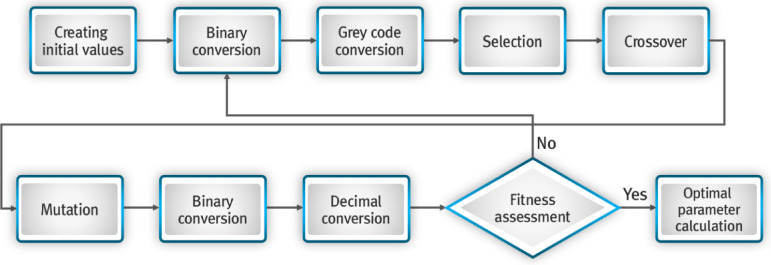
Process of calculating optimal parameters for tank model using genetic algorithm.

Using binary encoding directly during crossover can lead to the Hamming Cliff problem, in which small changes in parameter values may cause large changes in bit patterns. Therefore, in this study, we converted binary numbers into Gray codes, where the Hamming distance between adjacent numbers is 1, before performing crossover and mutation. Figure 5 shows the process of calculating the optimal parameters for a Four-Tank Model using a genetic algorithm.

#### Trend accuracy index

In rainfall−runoff models such as the Unit Hydrograph Method, the Storage Function, and the tank model, optimization methods such as genetic algorithms are used to improve model performance. In such cases, commonly used fitness functions in rainfall-runoff models include NSE and RMSE, as shown in Eqs. (12) and (13).

12 $$\:\mathrm{N}\mathrm{S}\mathrm{E}=1-\frac{\sum\:_{i=1}^{n}{({Q}_{obs,i}-{Q}_{sim,i})}^{2}}{\sum\:_{i=1}^{n}{({Q}_{obs,i}-{\stackrel{-}{Q}}_{obs,i})}^{2}}$$

13 $$\:\mathrm{R}\mathrm{M}\mathrm{S}\mathrm{E}=\sqrt{\frac{1}{n}\cdot\:\sum\:_{i=1}^{n}{({Q}_{sim,i}-{Q}_{obs,i})}^{2}}$$

$\:{Q}_{obs}$: *Observed dam inflow*.

$\:{Q}_{sim}$: *Simulated dam inflow*.

$\:{\stackrel{-}{Q}}_{obs}$: *Average observed dam inflow*.

In optimization methods, the objective functions are solely focused on minimizing the deviation between simulated values and observed values. Therefore, when using fitness functions such as NSE or RMSE, there are limitations in reproducing trends in observed dam inflow using the tank model. To address this issue, we propose a new objective function, the trend accuracy index (TAI), to better reproduce trends in dam inflow under future climate change scenarios. The index is defined in Eq. (14).

14 $$\begin{gathered} {\mathrm{TAI}} = 1 - \frac{{\sum {\left[ {\left| {\left\{ {\left( {OB_{j} - OB_{i} } \right) - (SIM_{j} - SIM_{i} )} \right\}} \right| \times \left\{ {1 + \alpha \: \cdot \:f((OB_{j} - OB_{i} ) \cdot \:(SIM_{j} - SIM_{i} ))} \right\}} \right]} }}{{\sum {\left| {(OB_{j} - OB_{i} )} \right|} }} \hfill \\ \;\;\;\;\;\;\;\;\;\;\;\;\;\;\;\;\;\;\;\;\;\;\;\;\;\;\;\;\;\;\;\;\;\;\;\;\;\;\;\;{\mathrm{j}} = {\mathrm{i}} + 1\:\:\:\:\:\:\:\:\:\:\:\:\:\: - \infty \: \le \:{\mathrm{TAI}} \le \:1 \hfill \\ \;\;\;\;\;\;\;\;\;\;\;\;\;\;\;\;\;\;\;\;\;\;\;\;\;\;\;\;\;\;\;\;\;\;\;\;\;\;\;\;\;\;\;\;\;\;f\left( x \right) = \frac{{1 - {\mathrm{tanh}}\left( {kx} \right)}}{2} \hfill \\ \;\;\;\;\;\;\;\;\;\;\;\;\;\;\;\;\;\;\;\;\;\;\;\;\;\;\;\;\;\;\;\;\;\;\;\;\;\;\;x = (OB_{j} - OB_{i} ) \cdot \:(SIM_{j} - SIM_{i} ) \hfill \\ \end{gathered}$$

$\:{OB}_{i}$: *Observed dam inflow at time i*.

$\:{OB}_{j}$: *Observed dam inflow at time j*.

$\:{SIM}_{i}$: *Simulated dam inflow at time i*.

$\:{SIM}_{j}$: *Simulated dam inflow at time j*.

$$\:\alpha\:$$: *Penalty parameter*.

$$\:k$$: *Steepness parameter*.

TAI calculates the difference between the change in observed values and the change in simulated values when the time point changes from i to j in continuous time-series data. The closer TAI is to 1, the higher the trend concordance rate between the simulated values and the observed values. Therefore, if the numerator $\:\sum\:\left|\left\{\left({OB}_{j}-{OB}_{i}\right)-({SIM}_{j}-{SIM}_{i})\right\}\right|$ is 0, TAI becomes 1, indicating that the simulated values calculated by the model perfectly replicate the trend of the observed values. In addition, when the signs of $\:({OB}_{j}-{OB}_{i})$ and $\:({SIM}_{j}-{SIM}_{i})$ in numerator $\:\sum\:\left|\left\{\left({OB}_{j}-{OB}_{i}\right)-({SIM}_{j}-{SIM}_{i})\right\}\right|$ are opposite, the sum of the deviations becomes larger, so applying the optimization method adjusts the parameters in the direction where the increase and decrease of the simulated values and observed values are consistent. In the numerator term $\:\left\{1+\alpha\:\cdot\:f\left(\right({OB}_{j}-{OB}_{i})\cdot\:({SIM}_{j}-{SIM}_{i}\left)\right)\right\}$, users can specify a penalty that is applied when the simulated and observed values change in opposite directions. In fact, as shown in Supplementary Fig. S1, when $\:k=500$, $\:f\left(x\right)$, which has a sigmoid shape, produces the result that the sign of $\:x$ is 0 if it is positive and 1 if it is negative, except in the case where $\:x$ is very close to 0. Therefore, since $\:x=({OB}_{j}-{OB}_{i})\cdot\:({SIM}_{j}-{SIM}_{i})$ here, the penalty can be adjusted conditionally based on whether the direction of increase or decrease between the simulated and observed values aligns.

If the sign of x is negative at a specific time point, the penalty term is activated. If $\:\alpha\:=1$ at this point, TAI receives double the penalty. Researchers using TAI can decide whether to apply penalties depending on the situation. If $\:\alpha\:$ is set to 0 and TAI is calculated without penalties, resulting in a value of 0.8, the trend concordance rate of the simulated values is 80% relative to the observed values.

In this study, we optimized 13 tank model parameters using a genetic algorithm and compared cases in which the fitness function was set to TAI ((1) $\:\alpha\:=1$, (2) $\:k=500$) or NSE, evaluating trend concordance with observed dam inflow.

## Application and results

### Cluster analysis using statistical characteristics of weather stations

**Fig. 6:**
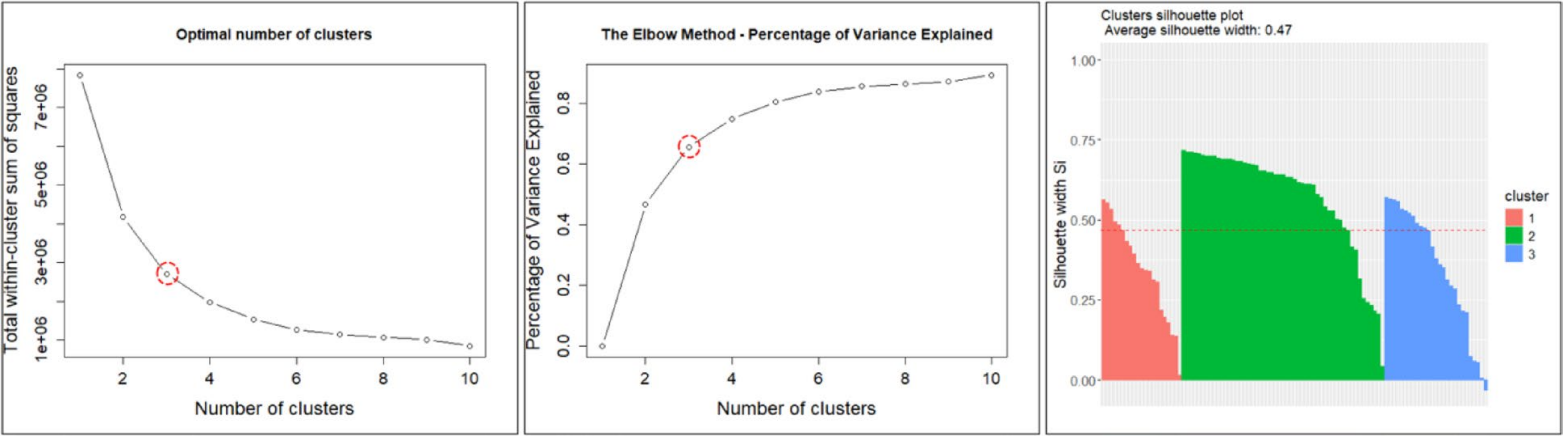
Selection of the optimal number of clusters for the weather stations (Left: WSS, Center: F-test, Right: Average Silhouette width).

**Fig. 7:**
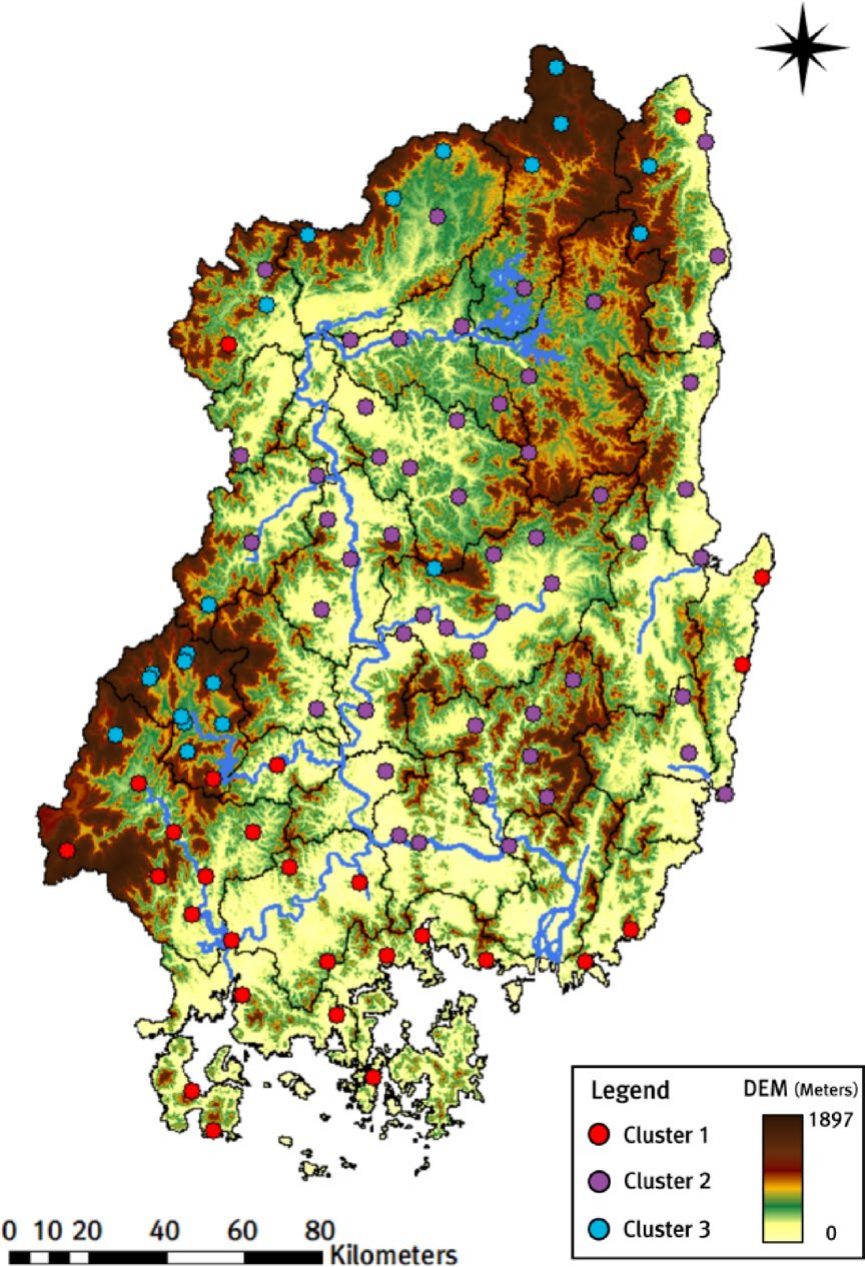
Distribution of weather stations by three clusters.

In this study, we performed cluster analysis using the K-means + + algorithm with indicator data from 101 weather stations in the Nakdong River basin for 2000–2019. The station-specific indicators consist of four variables: (1) elevation, (2) annual average rainfall, (3) average rainfall during the rainy season (June–September), and (4) average monthly maximum rainfall by years. In addition, we excluded location variables (latitude and longitude) because their effects are already reflected in the rainfall-related indicators; including them did not improve the preliminary clustering results. Before applying the K-means + + algorithm, we explored the optimal number of clusters ($\:k$) using the WSS, F-test, and Silhouette methods based on the four indicators. All three methods indicated that $\:k=3$ was the optimal number of clusters. In particular, the Silhouette method identified $\:k=3$ as the optimal number of clusters, with an average Silhouette width of 0.47. Figure 6 shows the WSS, F-test, and average silhouette width (for $\:k=3$), and Fig. 7 shows the spatial distribution of weather stations for $\:k=3$.

Figure 7 shows that Cluster 1 includes 27 stations; except for two stations in the northern Nakdong River basin, 25 stations are adjacent to the southern coastal area. Most stations in Cluster 2 are located in low-lying areas, whereas most stations in Cluster 3 are located in highland (mountainous) areas. Each weather station shows different rainfall characteristics depending on the cluster. Table 1 shows clear differences in indicator statistics among the clusters.

**Table 1:** Statistical values by indicators for the three clusters (2000–2019).

Classification	Statistical value	Average annual rainfall (mm)	Average rainfall during the rainy season (mm)	Average monthly maximum rainfall by years (mm)	Elevation (m)
Cluster 1 (*n* = 27)	Average	**1506.61**	**949.26**	**436.19**	**82.72**
	Maximum	1907.98	1224.92	561.30	478.65
	Minimum	1308.35	809.92	365.35	6.67
	Standard deviation	149.33	110.17	48.52	96.03
	Coefficient of variation	0.0991	0.1160	0.1112	1.1609
Cluster 2 (*n* = 53)	Average	**1115.40**	**732.76**	**335.55**	**89.06**
	Maximum	1263.45	863.60	403.02	316.39
	Minimum	956.00	642.00	285.02	3.94
	Standard deviation	81.98	53.17	27.58	67.79
	Coefficient of variation	0.0735	0.0725	0.0822	0.7612
Cluster 3 (*n* = 21)	Average	**1289.23**	**874.63**	**398.39**	**362.14**
	Maximum	1451.20	973.20	456.20	714.45
	Minimum	1174.08	786.29	346.95	173.01
	Standard deviation	72.79	46.76	29.66	152.36
	Coefficient of variation	0.0564	0.0534	0.0744	0.4207
All (*n* = 101)	Average	**1256.12**	**820.14**	**375.52**	**144.14**
	Maximum	1907.98	1224.92	561.30	714.45
	Minimum	956.00	642.00	285.02	3.94
	Standard deviation	194.89	119.43	56.06	148.95
	Coefficient of variation	0.1551	0.1456	0.1492	1.0333

When comparing the statistical values of the three indicators reflected in the cluster analysis—average annual rainfall, average rainfall during the rainy season, and average monthly maximum rainfall by years—the values were significantly higher in Cluster 1, followed by Cluster 3 and Cluster 2. Cluster 1, which has the highest statistical values for rainfall indicators, is located near the southern coastal region, and this geographical characteristic is reflected in its highest statistical values among the three clusters. In addition, Cluster 3, where most of the weather stations are located in the highlands, had significantly higher statistical values for rainfall indicators than Cluster 2, where the weather stations are located in the lowlands. Through this analysis, we can see that rainfall characteristics vary depending on the location and altitude of weather stations in the Nakdong River basin.

**Fig. 8:**
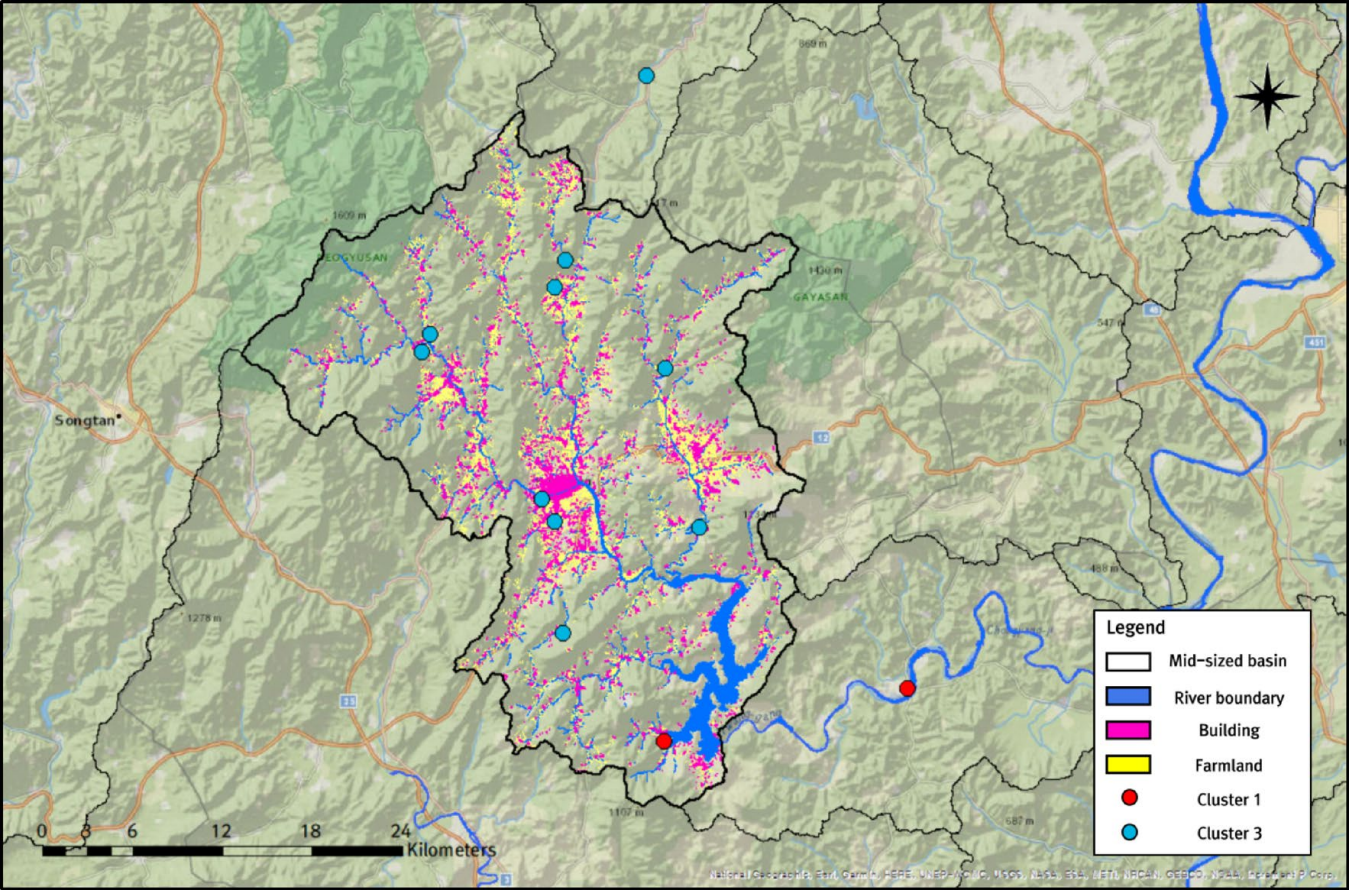
Distribution of 12 weather stations around the Hapcheon Dam basin by cluster. The map was created using ArcGIS Pro 3.6.1 (https://www.esri.com/en-us/arcgis/products/arcgis-pro/overview) with the National Geographic World Map basemap.

Furthermore, the Hapcheon Dam basin—selected as the pilot area for dam-inflow trend analysis in this study—shows clear, cluster-specific rainfall characteristics among surrounding weather stations. In the Hapcheon Dam basin, rainfall data were collected from 12 nearby weather stations to simulate inflow using the tank model. Given that over 70% of the basin area consists of forested regions, all 10 stations in Cluster 3 are located in high-elevation mountainous areas (Fig. 8). Moreover, the two stations in Cluster 1 are not adjacent to the coast; however, they are located very close to the dam in the lower basin, where large volumes of water are stored, and to the Hwang River, a national river. Indeed, we examined (1) annual average rainfall, (2) average rainfall during the rainy season (June–September), and (3) average monthly maximum rainfall by year. The averages for stations in Cluster 1 were 1434.46 mm, 990.98 mm, and 451.58 mm, whereas those for Cluster 3 were 1302.35 mm, 892.97 mm, and 414.91 mm. These results suggest that the Cluster 1 and Cluster 3 stations around the Hapcheon Dam basin have higher mean values for all indicators than the corresponding cluster means derived from the 101 stations in the Nakdong River basin, except for annual average rainfall in Cluster 1. Ultimately, the Hapcheon Dam basin features a gorge-like topography surrounded by mountains, which is advantageous for dam construction and securing storage capacity. In addition, the basin has maintained the water-supply capacity needed to secure water resources to date. The station-level rainfall characteristics derived from clustering can be integrated with dam inflow trend results to support efficient water-resources management.

### Homogeneity test of rainfall time-series data

Before performing trend analysis, three homogeneity tests (SNHT, Pettitt’s Test, BRT) were applied to rainfall time-series data from each weather station to select rainfall data that could provide reliable results for trend analysis. For monthly, seasonal, and annual rainfall series, we classified the data as ‘useful’ if they passed all three tests, ‘doubtful’ if they passed two tests, and ‘suspect’ if they passed one or none, thereby evaluating data reliability. Table 2 summarizes the results of homogeneity tests for monthly, seasonal, and annual rainfall data for 101 weather stations from 2000 to 2019. In Korea, the seasons are classified as spring (March to May), summer (June to August), autumn (September to November), and winter (December to February).

**Table 2:** Results of homogeneity tests for 101 weather stations (2000–2019).

Division	Useful	Doubtful	Suspect
January	78	22	1
February	101	0	0
March	99	2	0
April	99	1	1
May	74	22	5
June	94	7	0
July	100	1	0
August	101	0	0
September	98	3	0
October	0	15	86
November	83	18	0
December	99	2	0
Spring	97	4	0
Summer	57	20	24
Fall	92	5	4
Winter	101	0	0
Year	96	5	0

The homogeneity tests indicated that most weather stations passed for each period. However, in January, May, October, and summer, a substantial number of stations failed the homogeneity tests; in October, no stations were evaluated as ‘useful’. We found that irregular autumn typhoons in October caused substantial rainfall variability across the Nakdong River basin, which explains why no stations passed the homogeneity tests in October. In fact, when examining the annual occurrence of typhoons affecting the Nakdong River basin in October over the past 20 years, there were seven instances where four or more typhoons occurred in a single year. Therefore, we performed trend analyses only on rainfall series classified as ‘useful’ (i.e., those that passed all three homogeneity tests) to obtain reliable results.

### Analysis of past rainfall data trends and cluster characteristics

Table 3 shows the results of applying the modified MK test only to time-series data that passed all three homogeneity tests among 101 weather stations in the Nakdong River basin from 2000 to 2019.

**Table 3:** Number of weather stations showing monthly, seasonal, and annual trends that have passed homogeneity tests (2000–2019).

Division	The number of weather stations	Trend
January	8	Decrease (↓)
February	1	Increase (↑)
March	74	Increase (↑)
April	15	Increase (↑)
May	1	Decrease (↓)
June	31	Decrease (↓)
July	18	Decrease (↓)
August	17	Decrease (↓)
September	3	Decrease (↓)
October	0	−
November	2	Increase (↑)
December	11	Increase (↑)
Spring	3	Increase (↑)
Summer	27	Decrease (↓)
Fall	11	Increase (↑)
Winter	1	Increase (↑)
Year	4	Decrease (↓)

**Fig. 9:**
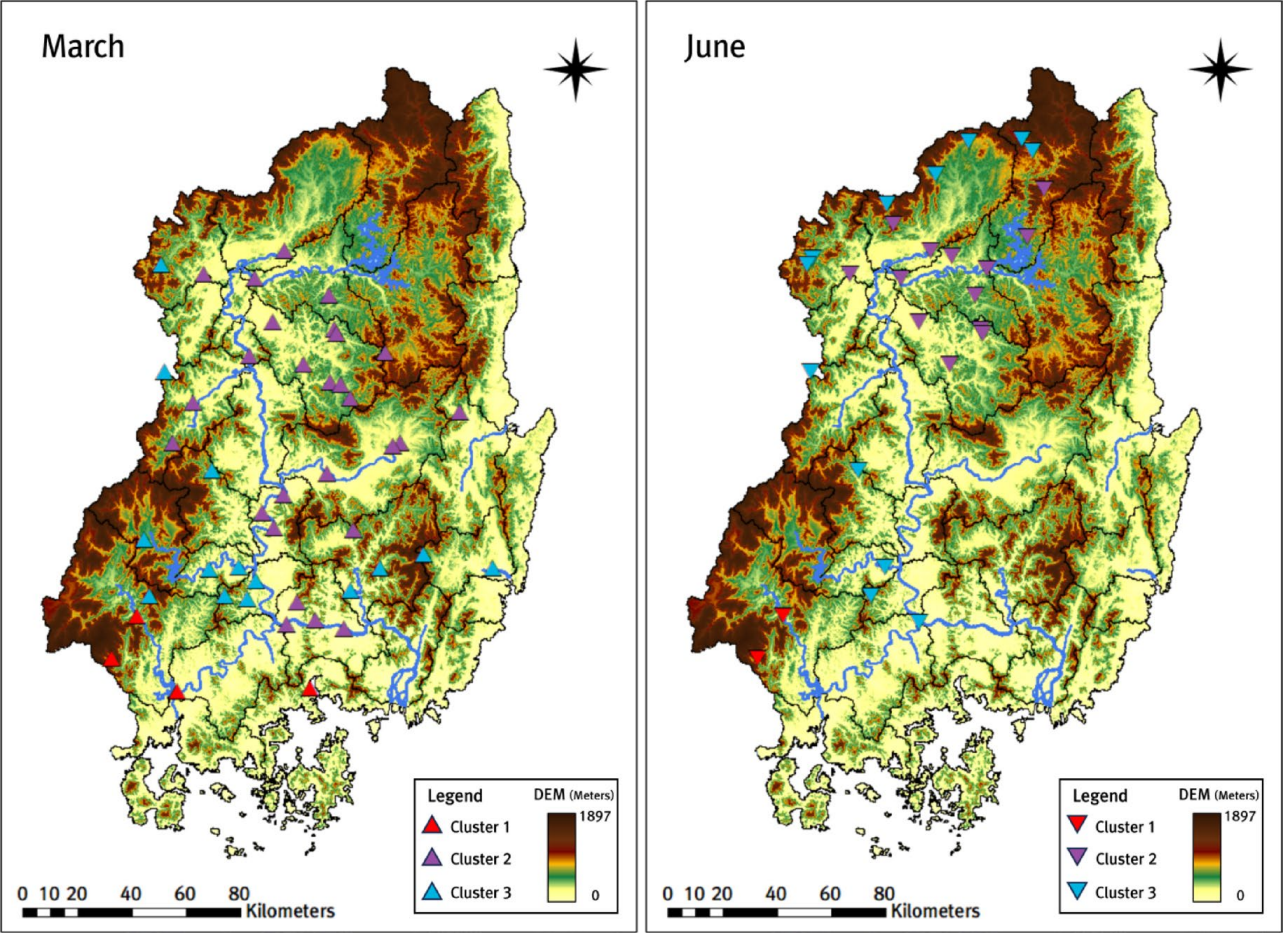
Distribution of increasing and decreasing trends in weather stations by clusters.

As a result of conducting trend analysis by month, season, and year from 2000 to 2019, all weather stations that showed trends during the same period showed the same direction (increase or decrease). Looking at the monthly trends, February, March, April, November, and December showed an increase, while January and May to September showed a decrease. Among the months with trends, the highest number of stations showing trends were recorded in March and June. Additionally, a notable feature was the continuous decrease in trends over five consecutive months from May to September, and this characteristic was also observed in the seasonal trends, specifically during the summer months of June to August. It is also notable that a decreasing trend was observed for five consecutive months from May to September, and this characteristic was also observed in the summer trend corresponding to June to August among seasonal trends. Finally, the annual trend also showed a decreasing trend, significantly influenced by the decrease in rainfall during the six months including January. In October, trends were detected at 94 of the 101 weather stations, and all of these trends were increasing. However, because no stations were classified as ‘useful’ in the homogeneity tests for October, the October trend results were deemed unreliable and were excluded from further discussion. Figure 9 shows the weather stations grouped by clusters, focusing on March and June, which exhibited the strongest trends in the trend analysis results.

In March, a total of 74 weather stations showed an increasing trend in rainfall, with the trend being particularly noticeable in Cluster 2, which is mainly located in low-lying areas, and Cluster 1, which is adjacent to the southern coastal region. In Cluster 3, except for two stations, no trend was observed in stations located in the upper reaches of the Nakdong River basin in highland areas, while the trend was primarily found in stations in the downstream highland areas. Conversely, in June, a decreasing trend was observed at a total of 31 weather stations, with a significant number of weather stations in Cluster 2 showing the trend in low-lying upstream areas of the Nakdong River basin. Additionally, in Cluster 3, a notable feature was that trends emerged in upstream highland weather stations where trends were almost absent in March. This analysis shows that the cluster-wise distribution of stations varies across periods in which trends emerge.

### Analysis of future rainfall data trends and cluster characteristics

Using the same methods for analyzing trends and clusters in past rainfall data, we also analyzed 101 weather stations for the future periods of 2021–2050 and 2051–2100. We analyzed two scenarios (SSP2-4.5 and SSP3-7.0), and Table 4 shows the monthly, seasonal, and annual trends for stations that have passed the homogeneity tests under the future climate scenarios.

**Table 4:** Number of weather stations showing monthly, seasonal, and annual trends (2021–2050 and 2051–2100).

Division	2021–2050				2051–2100			
	SSP2-4.5		SSP3-7.0		SSP2-4.5		SSP3-7.0	
	The number of weather stations	Trend	The number of weather stations	Trend	The number of weather stations	Trend	The number of weather stations	Trend
January	0	−	0	−	0	−	0	−
February	1	Increase (↑)	8	Increase (↑)	1	Decrease (↓)	0	−
March	0	−	0	−	0	−	0	−
April	0	−	0	−	36	Increase (↑)	0	−
May	0	−	0	−	0	−	0	−
June	0	−	0	−	0	−	0	−
July	12	Decrease (↓)	10	Increase (↑)	1	Decrease (↓)	0	−
August	0	−	0	−	0	−	11	Increase (↑)
September	0	−	0	−	0	−	0	−
October	0	−	0	−	0	−	0	−
November	0	−	0	−	0	−	0	−
December	0	−	0	−	0	−	14	Decrease (↓)
Spring	0	−	0	−	20	Increase (↑)	0	−
Summer	3	Decrease (↓)	8	Increase (↑)	0	−	2	Increase (↑)
Fall	0	−	0	−	0	−	8	Decrease (↓)
Winter	0	−	4	Increase (↑)	2	Decrease (↓)	0	−
Year	0	−	16	Increase (↑)	0	−	0	−

In the analysis of rainfall trends under future climate change scenarios, the number of weather stations showing trends by month, season, and year was lower than in the past. In particular, under the SSP2-4.5 scenario for the 2021–2050 period, trends were observed only in February, July, and summer, with only 16 weather stations showing trends. However, when comparing the trends observed during the same periods with those in the past, it was found that the trends in February, July, and summer were consistent. In the 2051–2100 SSP2-4.5 scenario, trends were observed at 60 weather stations in February, April, July, spring, and winter, and except for February, the trends in all periods were consistent with the past.

In the SSP3-7.0 scenario for 2021–2050, trends were observed in February, July, summer, winter, and annually. Both the number of weather stations where trends were detected and the number of observation periods during which trends were observed were significantly higher than those in the SSP2-4.5 scenario. Additionally, the trends showed increases at all time points, which is a key difference from the SSP2-4.5 scenario. In the 2051–2100 SSP3-7.0 scenario, trends were observed in August, December, summer, and autumn, and compared with the 2021–2050 scenario, the timing of the trends differed except for summer.

In the cluster analysis, based on the cluster analysis results derived from 101 weather stations in the past, the stations were classified into the same clusters in the future climate change scenarios, and the statistical values for each indicator were analyzed. Supplementary Tables S1–S4 summarize the statistical values for each indicator under the SSP2-4.5 and SSP3-7.0 scenarios for the periods 2021–2050 and 2051–2100.

When comparing the statistical values of past cluster-specific indicators based on the average annual rainfall with those of future climate change scenarios, the values were found to be largest in the order of SSP3-7.0, SSP2-4.5, and past (2000–2019). Notably, even under future scenarios, the magnitude of cluster-specific statistical values followed the same pattern as in the past, with Cluster 1, Cluster 3, and Cluster 2 ranking highest in that order.

However, when comparing the indicator sizes by cluster across future scenarios, several characteristics can be observed. First, for average annual rainfall, except for Cluster 1, which consists of weather stations located in the southern coastal region, the statistical values of all weather stations were larger for SSP3-7.0 than for SSP2-4.5. Additionally, except for the average monthly maximum rainfall, all statistical values for each indicator in the 2051–2100 period were larger than those in the 2021–2050 period.

The results of the analysis indicate that the statistical values of each indicator reflected in the cluster analysis generally show a proportional relationship with the magnitude of climate change risks. However, depending on the location and elevation of weather stations, the magnitude of statistical values may invert across scenarios, necessitating consideration of this phenomenon in water-resources management plans.

### Analysis of past trends in inflows to Hapcheon Dam

We also performed monthly, seasonal, and annual trend analyses using observed dam inflow data from the past (2000–2019) for the Hapcheon Dam basin, which was selected as a pilot area within the Nakdong River basin. As with the rainfall data, the trend analysis was performed only when the dam inflow data passed the three homogeneity tests, and a comparative analysis was performed to determine whether the results were consistent with the rainfall trends at the 12 weather stations around the Hapcheon Dam basin, as shown in Fig. 3. Table 5 summarizes the results of the trend analysis of past dam inflows to Hapcheon Dam.

**Table 5:** Homogeneity test and trend analysis of Hapcheon Dam inflow (2000–2019).

Division	Homogeneity test	Dam inflow trend	Rainfall trends at nearby stations	Number of nearby stations showing trends
January	Pass	−	−	−
February	Pass	Decrease (↓)	−	−
March	Pass	−	Increase (↑)	9
April	Pass	Increase (↑)	Increase (↑)	2
May	Pass	−	−	−
June	−	−	Decrease (↓)	4
July	Pass	Decrease (↓)	Decrease (↓)	6
August	Pass	−	−	−
September	Pass	−	−	−
October	−	−	−	−
November	Pass	−	−	−
December	−	−	Increase (↑)	1
Spring	Pass	−	Increase (↑)	1
Summer	−	−	Decrease (↓)	1
Fall	Pass	−	−	−
Winter	Pass	−	−	−
Year	Pass	−	Decrease (↓)	2

Looking at the results of the trend analysis of the inflow data for the Hapcheon Dam, we observed decreases in February and July and an increase in April. However, this differs from the rainfall trends observed at the 12 surrounding weather stations. Among the eight periods in which rainfall trends were detected, only April and July were consistent with the inflow trends; the other periods were not. In December, spring, and summer, only one station showed a rainfall trend, suggesting that rainfall trends may have had limited influence on Hapcheon Dam inflow during those periods. However, even in March, when nine of the 12 weather stations showed an increasing trend, no trend was observed in the inflow to Hapcheon Dam. Conversely, in February, no rainfall trends were observed at nearby stations, but Hapcheon Dam inflow showed a decreasing trend. Based on these analysis results, it can be inferred that the trends in dam inflow and surrounding rainfall do not always align, and that factors such as temperature, humidity, land cover, and domestic wastewater discharged from urban areas upstream in the basin also have a complex influence on the trends in dam inflow.

#### Realization of past inflow trends to Hapcheon Dam using a four-tank model

In this study, in addition to analyzing the trend in historical Hapcheon Dam inflow, we performed trend analyses for two future periods (2021–2050 and 2051–2100) under SSP2-4.5 and SSP3-7.0. To accurately estimate the inflow trend for the Hapcheon Dam for future periods, we optimized the Four-Tank Model using the newly developed TAI and PET-CF. To evaluate how TAI and PET-CF improve inflow-trend accuracy, we compared three optimization cases for the Four-Tank Model using a genetic algorithm: (1) TAI + PET-CF (Case 1), (2) NSE + PET-CF (Case 2), and (3) NSE only (Case 3). Table 6 summarizes the results of optimizing the Four-Tank Model parameters using a genetic algorithm for the three cases.

**Table 6:** Optimization results for the tank model parameters using a genetic algorithm.

Division	Weight	Case 1 (use TAI, $\:{\alpha\:}_{modi}$)	Case 2 (use NSE, $\:{\alpha\:}_{modi}$)	Case 3 (use only NSE)
Infiltration coefficients for each tank	$\:{A}_{0}$	0.052	0.094	0.654
	$\:{B}_{0}$	1.754	0.314	1.276
	$\:{C}_{0}$	0.020	0.192	1.452
Runoff coefficients for each tank	$\:{A}_{1}$	0.320	0.636	0.962
	$\:{A}_{2}$	0.468	0.180	0.576
	$\:{B}_{1}$	1.104	1.864	1.660
	$\:{C}_{1}$	1.632	1.584	2.026
	$\:{D}_{1}$	0.162	0.612	1.256
Heights of runoff outlets for each tank	$\:{HA}_{1}$	2	80	0
	$\:{HA}_{2}$	98	304	1138
	$\:HB$	1634	98	0
	$\:HC$	116	596	0
Potential-evapotranspiration correction factor	$\:{\alpha\:}_{modi}$	0.433	0.363	−

**Fig. 10:**
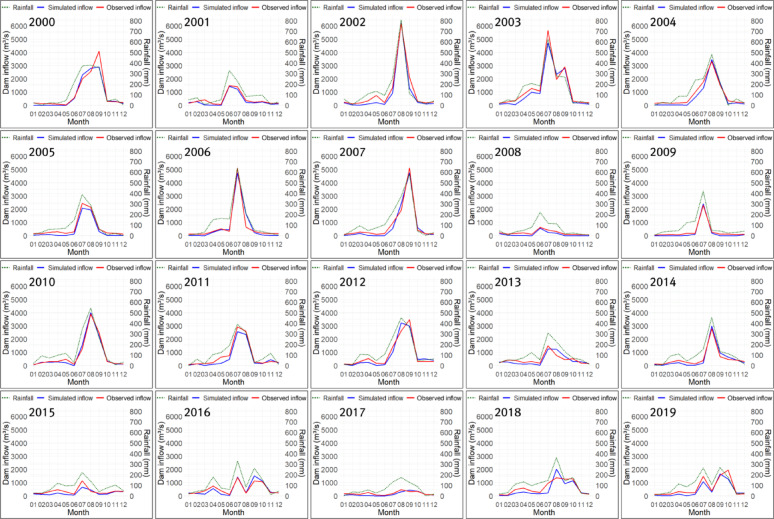
Simulated results of monthly inflow at Hapcheon Dam during 2000–2019 (Case 1).

**Fig. 11:**
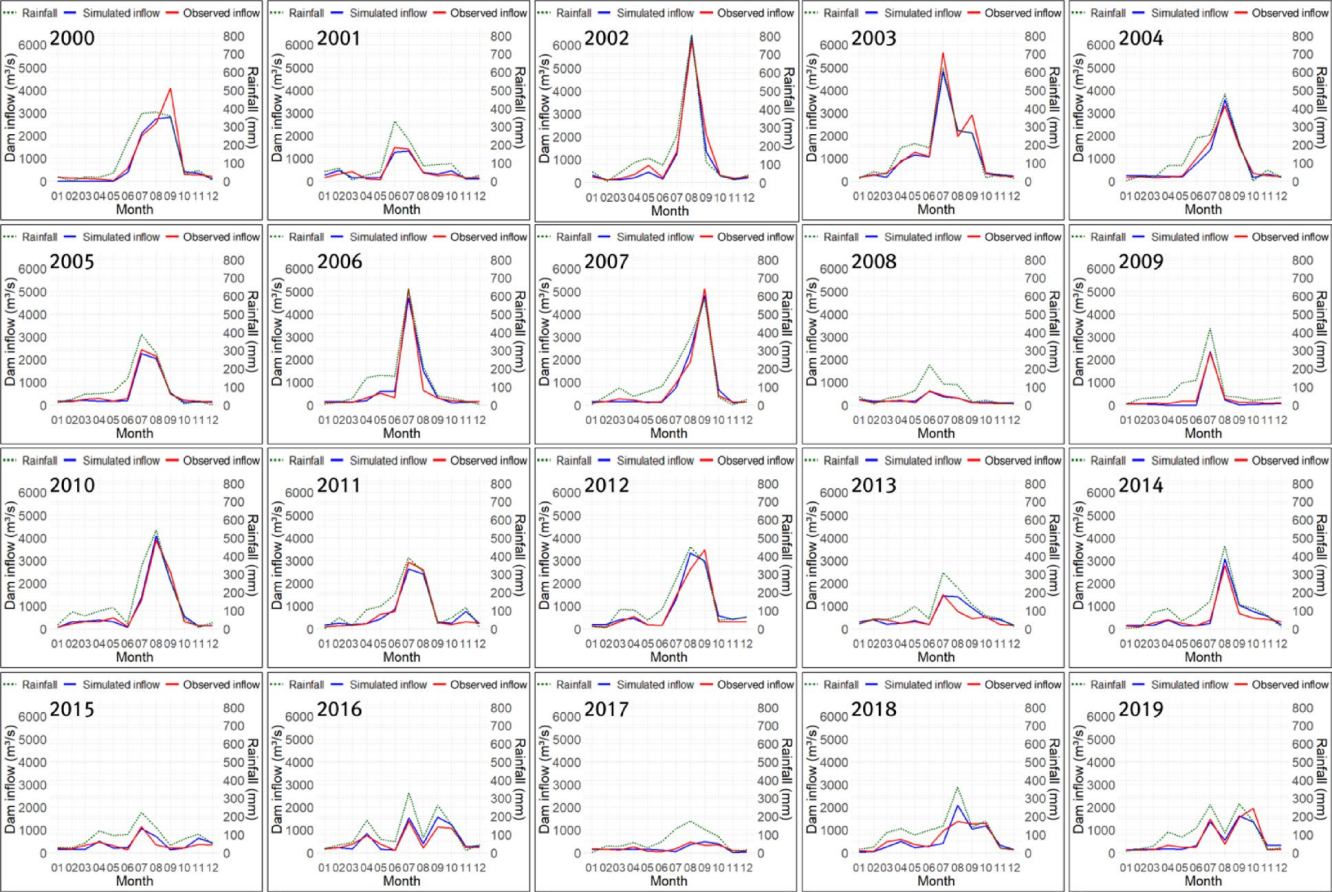
Simulated results of monthly inflow at Hapcheon Dam during 2000–2019 (Case 2).

**Fig. 12:**
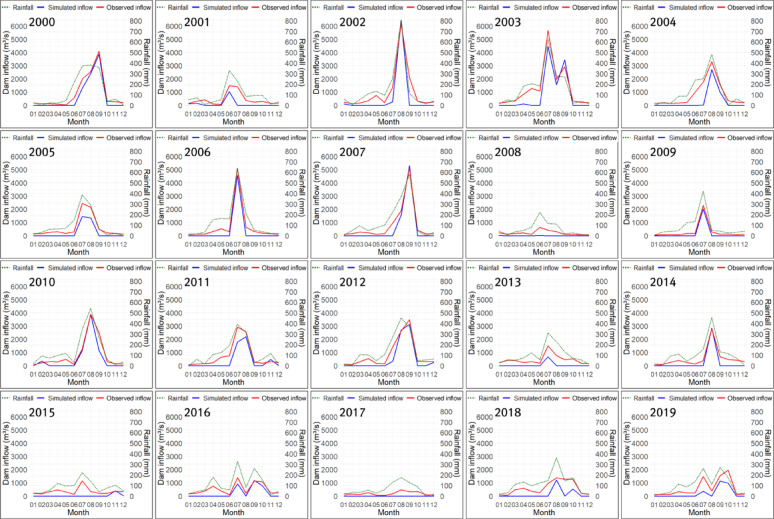
Simulated results of monthly inflow at Hapcheon Dam during 2000–2019 (Case 3).

The optimized parameters differed substantially between Cases 1 and 2, depending on the fitness function used in the genetic algorithm. In particular, in Case 1, which used TAI as the fitness function, the PET-CF $\:{\alpha\:}_{modi}$ and the height of runoff outlet $\:HB$ of the second tank were larger than those in Case 2, while the infiltration coefficient $\:{A}_{0}$ of the first tank was smaller than that in Case 2. Through this, it can be inferred that Case 1 was optimized toward reducing the size of the dam inflow calculated by the tank model compared to Case 2, while improving trend accuracy. In Case 3, since the PET-CF was not applied, the infiltration and runoff coefficients for each tank were calculated to be very large, and the heights of runoff outlets for each tank were all calculated to be 0, except for the upper discharge opening of the first tank. This suggests that optimization was conducted in Case 3 to mitigate the issue of underestimating dam inflow due to potential evapotranspiration. Figures 10, 11 and 12 show the monthly inflow simulation results for the Hapcheon Dam from 2000 to 2019, obtained by applying the parameters optimized by the genetic algorithm to the Four-Tank Model, for each case.

As shown in the figures, the monthly simulated inflow in Case 1 was smaller than that in Case 2, resulting in a larger deviation from the observed inflow; however, its trend was more similar to the observed trend. In Case 3, due to the effect of not applying the PET-CF, the simulated dam inflow was calculated to be zero, except for months with high rainfall. Table 7 compares the results of the trend analysis of the past simulated dam inflows for each case calculated using the tank model with the observed dam inflow trends. We assessed trend-reproduction accuracy for each case by considering the observed inflow trends (2000–2019) and the consistency of homogeneity test results.

**Table 7:** Comparison of the accuracy of trends in dam inflows for each case.

Division	Trend of Case 1 (homogeneity test)	Trend of CASE 2 (homogeneity test)	Trend of Case 3 (homogeneity test)	Trend of observed dam inflows (homogeneity test)
January	− (Pass)	− (Pass)	− (−)	− (Pass)
February	− (Pass)	− (Pass)	− (Pass)	Decrease (↓) (Pass)
March	Increase (↑) (Pass)	− (Pass)	− (Pass)	− (Pass)
April	Increase (↑) (Pass)	− (Pass)	− (Pass)	Increase (↑) (Pass)
May	− (Pass)	− (Pass)	− (−)	− (Pass)
June	− (−)	Decrease (↓) (Pass)	− (−)	− (−)
July	Decrease (↓) (Pass)	− (Pass)	− (Pass)	Decrease (↓) (Pass)
August	− (Pass)	− (Pass)	− (Pass)	− (Pass)
September	− (Pass)	− (Pass)	− (Pass)	− (Pass)
October	− (−)	− (−)	− (−)	− (−)
November	− (−)	− (−)	− (Pass)	− (Pass)
December	− (−)	− (Pass)	− (Pass)	− (−)
Spring	− (Pass)	− (Pass)	− (Pass)	− (Pass)
Summer	− (−)	− (−)	Decrease (↓) (Pass)	− (−)
Fall	− (Pass)	− (Pass)	− (Pass)	− (Pass)
Winter	− (−)	− (Pass)	− (Pass)	− (Pass)
Year	− (Pass)	− (Pass)	Decrease (↓) (Pass)	− (Pass)
Accuracy of trend	13 out of 17 (76.4%)	10 out of 17 (58.8%)	9 out of 17 (52.9%)	−
TAI	0.6154	0.5451	0.4121	−
NSE	0.9401	0.9504	0.7831	−
RMSE	237.6106	216.1882	452.0993	−

As a result of comparing the accuracy of monthly, seasonal, and annual trends for each case based on observed dam inflows, Case 1, which applied TAI and PET-CF, was found to have the highest accuracy at 76.4%. On the other hand, the accuracy of Cases 2 and 3 was 58.8% and 52.9%, respectively, which was up to 23.5% lower than that of Case 1. Upon conducting a detailed analysis of the results, several characteristics were identified. First, even in Cases 2 and 3, where TAI was not set as the fitness function, TAI was calculated in proportion to the trend accuracy. This shows that TAI accurately implemented the trend accuracy of long-term hydrological time-series data. Second, when comparing Case 2 and Case 3, the PET-CF plays a very important role in improving the accuracy of the dam inflow amount and trend depending on whether it is applied or not. In particular, the RMSE in Case 3 was more than twice as large as that in Case 2, and TAI was calculated to be low, confirming that both TAI and the PET-CF are necessary to obtain optimal trend analysis results for dam inflow. Third, even when optimization is performed with TAI set as the fitness function as in Case 1, when a comparison of TAI, NSE, and RMSE with those of Case 2, where NSE was set as the fitness function, TAI is 0.0703 higher, while there is no significant difference in NSE and RMSE. Due to these characteristics, Case 1 showed a high concordance rate with observed dam inflows not only in trend analysis but also in homogeneity testing. Therefore, it can be concluded that using TAI and the PET-CF is the correct approach for improving the accuracy when performing trend analysis on long-term hydrological time-series data, such as the simulated dam inflows calculated using the tank model.

Finally, the optimized tank models for each case were verified using observed dam inflow data for 2020–2024. Table 8 compares the TAI, NSE, and RMSE for each case for 2020–2024.

**Table 8:** Verification of tank model accuracy by case (2020–2024).

Division	Case 1	Case 2	Case 3
TAI	0.5878	0.5507	0.1443
NSE	0.9369	0.9373	0.7950
RMSE	281.8118	280.7203	507.9803

The verification results also showed that Case 1 had a TAI value that was 0.0371 higher than Case 2, while there was no significant difference in NSE and RMSE performance. In contrast, Case 3 demonstrated significantly lower performance in TAI, NSE, and RMSE compared to the other cases. Based on these findings, the tank model in Case 1 was confirmed to be the most suitable for analyzing trends in the Hapcheon Dam inflow considering future climate change scenarios.

#### Trend analysis of future dam inflow into Hapcheon Dam

When calculating the Hapcheon Dam inflows for the SSP2-4.5 and SSP3-7.0 scenarios during the periods 2021–2050 and 2051–2100, we used scenario-specific statistical data provided by the Climate Information Portal together with the tank model in Case 1. We then performed homogeneity testing and trend analysis for dam inflows for each scenario. Supplementary Tables S5 and S6 summarize trend-analysis results for Hapcheon Dam inflow and rainfall at nearby stations for 2021–2050 and 2051–2100 under each climate scenario.

When examining the trends in dam inflows under climate change scenarios, an increasing trend was observed in November under the SSP2-4.5 scenario for 2051–2100. The results of this trend analysis are highly reliable as they were derived using the tank model in Case 1, which greatly improved accuracy by applying both TAI and PET-CF. However, no such trend was observed at other times, and in fact, very few monthly, seasonal, or annual trends were observed in rainfall data from weather stations around the Hapcheon Dam basin. Specifically, among the 12 stations, trends appeared at four or more stations only in August (increase) and December (decrease) under SSP3-7.0 during 2051–2100 (Supplementary Figs. S2 and S3). Furthermore, as shown in Table 5, there were many cases where the trends in observed dam inflow data did not match the trends in rainfall at nearby weather stations. Therefore, the mismatch between dam inflow and rainfall trends likely reflects the combined effects of non-rainfall factors. Finally, the proposed methodology can help inform proactive measures for efficient water-resources management under future climate scenarios in the Hapcheon Dam basin.

## Discussion

### Comparison of differences in the implementation of dam inflow trends using fitness functions

**Fig. 13:**
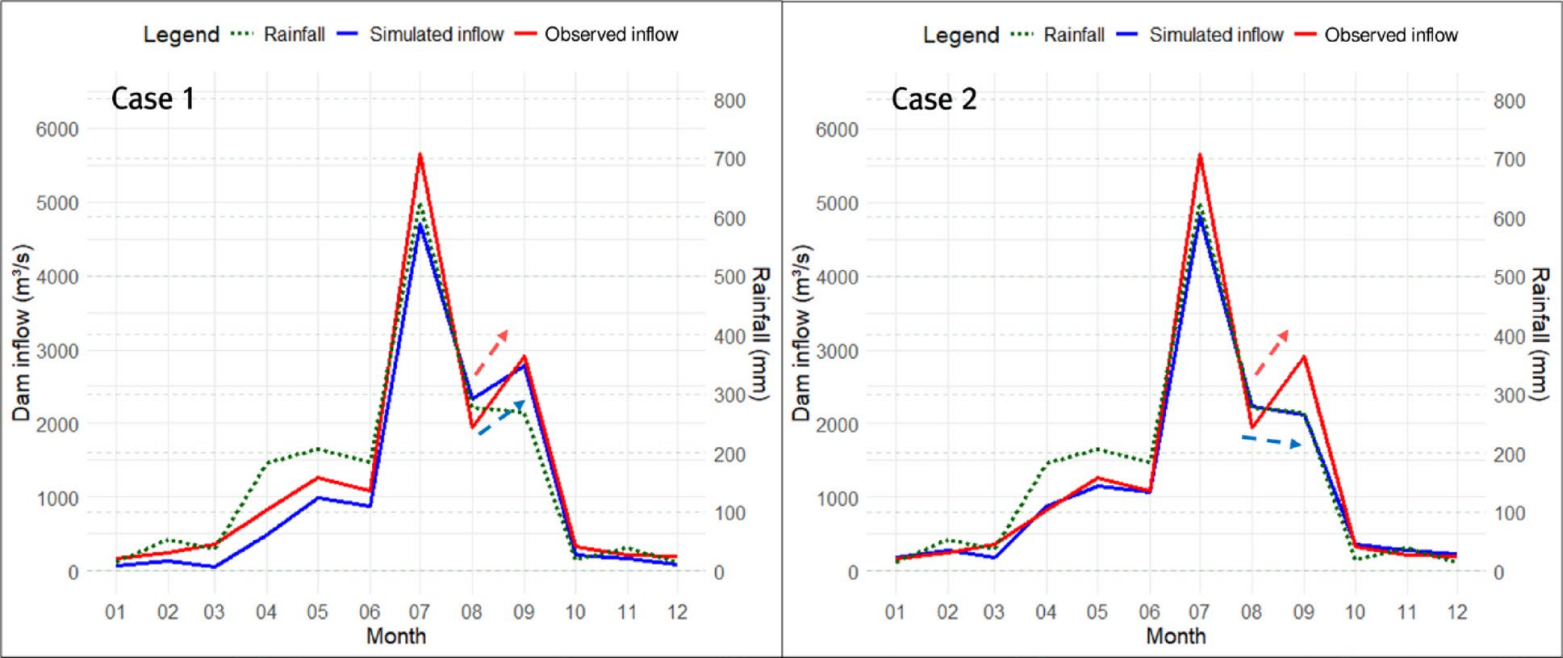
Comparison of trends in Hapcheon Dam inflow for 2003.

In this study, we compared the accuracy of trends depending on whether TAI or NSE was used as the fitness function in the parameter optimization of a Four-Tank Model using a genetic algorithm. Figure 13 compares Case 1, which applies TAI and PET-CF, with Case 2, which uses NSE instead of TAI; both cases are based on 2003 data.

**Fig. 14:**
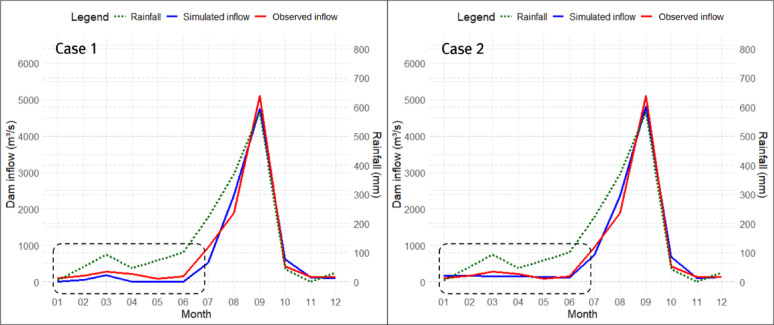
Comparison of trends in Hapcheon Dam inflow for 2007.

When comparing the January–December simulated dam inflows for each case, the monthly deviation between observed and simulated inflows is smaller for Case 2 than for Case 1. However, when comparing deviations and trends against the observed inflow, the largest difference occurred between August and September. In Case 1, the simulated inflow shows an increasing trend that matches the observed inflow, whereas Case 2 shows a decreasing trend. This difference has two main causes: (1) rainfall and observed inflow trends were opposite during that period, and (2) the optimization process differs depending on whether TAI or NSE is used as the fitness function. In this study, TAI receives a penalty of 2 when the trends of observed dam inflow and simulated dam inflow are opposite. Therefore, when the trends are opposite and a large deviation may occur, such as from August to September, the trend should be matched to minimize the penalty. Conversely, in Case 2, which uses NSE, the focus is on minimizing monthly dam inflow deviation. Therefore, even if observed and simulated trends are opposite during the August–September transition, minimizing deviations in other periods can be more beneficial, leading to the Case 2 outcome. This contrast is also evident in Fig. 14, which presents Hapcheon Dam inflow in 2007.

When comparing the simulated dam inflows from Case 1 and Case 2 derived from the tank model in 2007, the largest difference occurred between January and June. In Case 1, the trend of simulated dam inflows from January to May was consistent with observed dam inflows. However, while rainfall increased from April to June, observed dam inflows alternated between decreasing and increasing trends. Therefore, in order to minimize penalties, the parameters for the simulated dam inflows in Case 1 during this period were adjusted so that the inflow slope for May and June was zero. On the other hand, in Case 2, the simulated dam inflows from January to June showed a slope close to the x-axis without significant fluctuations within the range of 117.8–156.6 $\:{m}^{3}/s$. As a result, Case 2 showed a significant reduction in monthly inflow deviation over the six-month period compared with Case 1, but its trend accuracy was much lower. These results indicate that conventional objective functions (e.g., NSE and RMSE) are effective for reducing errors between simulated and observed values, but they are limited in improving trend accuracy simultaneously. Therefore, when performing trend analysis of long-term hydrological time-series data, such as future simulated dam inflows estimated using the tank model, optimizing the model parameters using TAI and PET-CF as in Case 1 can both reduce errors and improve trend accuracy. Linking the improved trend analysis results for future dam inflows with trend analysis of rainfall and cluster analysis results can contribute to efficient basin-level water-resources management that accounts for climate change.

### Limitations of the trend accuracy index

Although TAI is useful for improving trend accuracy when optimizing rainfall−runoff model parameters (e.g., the tank model), it has several limitations. Supplementary Fig. S4 shows a graph of observed dam inflows and simulated dam inflows from January to May.

In the figure, the observed dam inflow from January to May increased steadily by 100 $\:{m}^{3}/s$ each month from 100 $\:{m}^{3}/s$ to 500 $\:{m}^{3}/s$, and the simulated dam inflow also increased by the same amount each month from 0 $\:{m}^{3}/s$ to 400 $\:{m}^{3}/s$. Although the monthly inflow has a constant error of 100 $\:{m}^{3}/s$, substituting these values into Eq. (14) yields TAI = 1. This issue arises because TAI focuses on the accuracy of the trend. If the slopes of the observed dam inflow and the simulated dam inflow are the same, TAI determines that the trend is 100% consistent even if there is an error between the two data sets. Second, Eq. (14) also has the issue that TAI diverges to infinity when the denominator $\:\sum\:\left|({OB}_{j}-{OB}_{i})\right|$ approaches 0. However, the limitations mentioned above only occur with short-term hydrological time-series data, and these issues are not significantly highlighted in analyses using long-term hydrological time-series data, as in this study. In fact, since the tank model uses rainfall and evapotranspiration as independent variables, when using long-term hydrological time-series data rather than short-term data, it is rare for the slopes of the observed and simulated inflows to be identical at all time points, as shown in Supplementary Fig. S4. Additionally, in normal long-term hydrological time-series data, the denominator $\:\sum\:\left|({OB}_{j}-{OB}_{i})\right|$ in Eq. (14) cannot be zero, so this issue also does not become prominent. Therefore, TAI is a suitable objective function for trend analysis using long-term rather than short-term hydrological time-series data, and its use should be determined by considering these points.

In this study, as shown in Supplementary Fig. S1, a sigmoid-shaped $\:f\left(x\right)$ was applied to TAI to impose a penalty proportional to the size of $\:\alpha\:$ when the direction of change in the simulated values is opposite to that in the observed values. At this point, if the sign of $\:x$ in $\:f\left(x\right)$ is positive, $\:f\left(x\right)$ equals 0, and if it is negative, $\:f\left(x\right)$ equals 1. However, if $\:x$ is 0, $\:f\left(x\right)$ equals 0.5. In Eq. (14), this occurs when both $\:({OB}_{j}-{OB}_{i})$ and $\:({SIM}_{j}-{SIM}_{i})$ are 0 or either of them is 0. If both are 0, the numerator $\:\left({OB}_{j}-{OB}_{i}\right)-({SIM}_{j}-{SIM}_{i})$ is also 0, so the penalty application is irrelevant. However, if only one is 0, a 1.5-fold penalty is applied when $\:\alpha\:=1$ at that point. This phenomenon occurs because the penalty-imposing function $\:f\left(x\right)$ was added when developing the performance metric TAI. This is a mathematical result inherent in developing TAI as a function, so it cannot be logically deemed either unconditionally valid or invalid. However, in this study, since the trend accuracy improved significantly compared to NSE when using TAI, we retained this characteristic of TAI. If, in another study, researchers wish to modify the condition under which a 1.5-fold penalty is applied when $\:\alpha\:=1$ and $\:x=0$ in the TAI calculation, adding a separate conditional statement for cases where $\:x=0$ can improve TAI so that it better aligns with the research objectives and required performance.

## Conclusions

In this study, we performed cluster and trend analyses for the historical period (2000–2019) and for future climate scenarios (2021–2050 and 2051–2100) using rainfall data from 101 stations in the Nakdong River basin and Hapcheon Dam inflow data. Using the modified MK test, we identified monthly, seasonal, and annual trends in rainfall and dam inflow only from time series that passed all three homogeneity tests. To simulate dam inflow, we optimized the Four-Tank Model parameters using a genetic algorithm with TAI and PET-CF to improve trend reproduction. Based on these analyses, we derived the following implications.

The first implication concerns rainfall characteristics by station location and elevation, derived from K-means + + clustering of the 101 stations in the Nakdong River basin. The indicators—elevation, average annual rainfall, average rainfall during the rainy season, and the average monthly maximum rainfall by years—followed the pattern Cluster 1 > Cluster 3 > Cluster 2 in both historical and future periods. Overall, rainfall characteristics varied with station elevation and location; for example, Cluster 3 (high-elevation stations) showed higher indicator values than Cluster 2 (low-lying stations). This pattern is also supported by Cluster 1, whose coastal-proximate stations exhibited higher indicator values than inland clusters.

The second implication concerns data reliability in trend analysis. To improve the reliability of rainfall and dam-inflow trend analysis, we restricted the analysis to time series that passed all three homogeneity tests. Consequently, October—when autumn typhoons frequently occurred during 2000–2019—was excluded from the trend assessment because none of the stations were classified as ‘useful’ after the homogeneity tests. If trends during the excluded period must be evaluated, they may be examined using long-term hydrological time-series plots and the modified MK test, regardless of the homogeneity-test outcomes. Additionally, trend analyses that exclude typhoon-affected periods could be conducted to assess how these events influence the trend results.

The third implication concerns monthly, seasonal, and annual trends in rainfall and dam inflow. Trend analysis of historical rainfall (2000–2019) showed increasing trends in February–April and November–December, and decreasing trends in January and May–September. This trend was also observed in the seasonal and annual trends. In addition, weather stations that showed trends in the same month, season, and year showed consistent trend directions. However, when comparing the dam inflow trends in the Hapcheon Dam basin in the past and future with the rainfall trends at nearby weather stations, discrepancies were found in certain periods. These results indicate that dam inflow and rainfall trends in the Hapcheon Dam basin do not always match. This discrepancy likely reflects the combined effects of rainfall as well as meteorological, geomorphological, and other external drivers of inflow.

The fourth implication concerns the usefulness of TAI and PET-CF for reproducing observed dam-inflow trends in Four-Tank Model simulations. In this study, we compared model performance across cases with and without TAI and PET-CF, and the trend accuracy of Case 1, which applied both methods, was superior to that of the other cases. In particular, TAI was highly useful in improving trend accuracy compared to NSE and RMSE, which focus solely on reducing the discrepancy between simulated and observed values in existing rainfall-runoff models. Therefore, the results suggest that using TAI as a benchmark can be an effective approach for improving accuracy in trend analysis of long-term hydrological time series.

The results of the trend and cluster analysis of monthly, seasonal, and annual rainfall and dam inflow presented in this study can be used for efficient water-resources management in the Nakdong River basin, considering future climate change scenarios. In particular, given that TAI improved trend-analysis accuracy in this study, future work could assess its suitability in other basins and investigate how the $\:\alpha\:$ value in TAI influences trend-analysis accuracy.

## Supplementary Information

Below is the link to the electronic supplementary material.

Supplementary Material 1

## Data Availability

The datasets generated and/or analyzed during the current study are available from the corresponding author on reasonable request.
