# Supplementary material for: Trend analysis of dam inflow data using the trend accuracy index and the potential-evapotranspiration correction factor
Source: Sci Rep. 2026 Feb 20;16:10040. doi: 10.1038/s41598-026-40225-y (PMC13022278; doi:10.1038/s41598-026-40225-y)
Supplement: Supplementary file 1 — Supplementary Material 1 [file 41598_2026_40225_MOESM1_ESM.docx]

**Supplementary Materials for**

**Trend analysis of dam inflow data using the Trend Accuracy Index**

**and the Potential-Evapotranspiration Correction Factor**

Won-joon Wang^1^ · Hung Soo Kim^2*^

^1^ Gyeonggi Research Institute, Suwon, South Korea

^2^ Department of Civil Engineering, Inha University, Incheon, South Korea

*** *Corresponding author.* E-mail: sookim@inha.ac.kr

**Supplementary material comprises:**

Table S1: Statistical values by indicators for the three clusters (SSP2-4.5, 2021–2050).

Table S2: Statistical values by indicators for the three clusters (SSP2-4.5, 2051–2100).

Table S3: Statistical values by indicators for the three clusters (SSP3-7.0, 2021–2050).

Table S4: Statistical values by indicators for the three clusters (SSP3-7.0, 2051–2100).

Table S5: Homogeneity test and trend analysis of Hapcheon Dam inflow (2021–2050).

Table S6: Homogeneity test and trend analysis of Hapcheon Dam inflow (2051–2100).

Fig. S1: Changes in the $\boldsymbol{f}\left( \boldsymbol{x} \right)$ graph according to $\boldsymbol{k}$.

Fig. S2: Distribution of the 4 weather stations that show an increasing trend in the Hapcheon Dam Basin. The map was created using ArcGIS Pro 3.6.1 (https://www.esri.com/en-us/arcgis/products/arcgis-pro/overview) with the National Geographic World Map basemap.

Fig. S3: Distribution of the 10 weather stations that show a decreasing trend in the Hapcheon Dam Basin. The map was created using ArcGIS Pro 3.6.1 (https://www.esri.com/en-us/arcgis/products/arcgis-pro/overview) with the National Geographic World Map basemap.

Fig. S4: Example of observed and simulated dam inflows (January–May).

Table S1: Statistical values by indicators for the three clusters (SSP2-4.5, 2021–2050).

| Classification | Statistical value | Average annual rainfall(mm) | Average rainfall during the rainy season(mm) | Average monthly maximum rainfall(mm) | Elevation(m) |
| --- | --- | --- | --- | --- | --- |
| Cluster 1  (n = 27) | Average | **1578.31** | **1027.08** | **427.45** | **82.72** |
|  | Maximum | 2019.69 | 1351.89 | 567.42 | 478.65 |
|  | Minimum | 1338.89 | 838.47 | 357.24 | 6.67 |
|  | Standard deviation | 176.07 | 123.49 | 52.80 | 96.03 |
|  | Coefficient of variation | 0.1115 | 0.1202 | 0.1235 | 1.1609 |
| Cluster 2  (n = 53) | Average | **1155.15** | **771.32** | **322.92** | **89.06** |
|  | Maximum | 1444.22 | 986.68 | 420.16 | 316.39 |
|  | Minimum | 981.13 | 658.05 | 275.86 | 3.94 |
|  | Standard deviation | 111.41 | 72.04 | 27.96 | 67.79 |
|  | Coefficient of variation | 0.0964 | 0.0933 | 0.0865 | 0.7612 |
| Cluster 3  (n = 21) | Average | **1388.02** | **946.22** | **394.35** | **362.14** |
|  | Maximum | 1598.61 | 1081.97 | 443.48 | 714.45 |
|  | Minimum | 1221.95 | 826.15 | 344.69 | 173.01 |
|  | Standard deviation | 100.40 | 64.64 | 24.61 | 152.36 |
|  | Coefficient of variation | 0.0723 | 0.0683 | 0.0624 | 0.4207 |
| All  (n = 101) | Average | **1316.69** | **876.06** | **365.72** | **144.14** |
|  | Maximum | 2019.69 | 1351.89 | 567.42 | 714.45 |
|  | Minimum | 981.13 | 658.05 | 275.86 | 3.94 |
|  | Standard deviation | 223.48 | 143.34 | 58.58 | 148.95 |
|  | Coefficient of variation | 0.1697 | 0.1636 | 0.1601 | 1.0333 |

Table S2: Statistical values by indicators for the three clusters (SSP2-4.5, 2051–2100).

| Classification | Statistical value | Average annual rainfall(mm) | Average rainfall during the rainy season(mm) | Average monthly maximum rainfall(mm) | Elevation(m) |
| --- | --- | --- | --- | --- | --- |
| Cluster 1  (n = 27) | Average | **1669.71** | **1051.92** | **430.14** | **82.72** |
|  | Maximum | 2096.41 | 1359.29 | 550.56 | 478.65 |
|  | Minimum | 1408.04 | 916.47 | 374.32 | 6.67 |
|  | Standard deviation | 179.5 | 113.6 | 44.7 | 96.03 |
|  | Coefficient of variation | 0.1075 | 0.1079 | 0.1039 | 1.1609 |
| Cluster 2  (n = 53) | Average | **1237.45** | **808.75** | **327.47** | **89.06** |
|  | Maximum | 1524.66 | 1004.71 | 406.87 | 316.39 |
|  | Minimum | 1042.65 | 696.96 | 277.32 | 3.94 |
|  | Standard deviation | 115.69 | 68.6 | 30.68 | 67.79 |
|  | Coefficient of variation | 0.0934 | 0.0848 | 0.0936 | 0.7612 |
| Cluster 3  (n = 21) | Average | **1462.04** | **972.14** | **388.2** | **362.14** |
|  | Maximum | 1734.98 | 1130.14 | 462.65 | 714.45 |
|  | Minimum | 1268.28 | 855.8 | 344.13 | 173.01 |
|  | Standard deviation | 107.19 | 67.16 | 28.36 | 152.36 |
|  | Coefficient of variation | 0.0733 | 0.069 | 0.073 | 0.4207 |
| All  (n = 101) | Average | **1399.7** | **907.73** | **367.54** | **144.14** |
|  | Maximum | 2096.41 | 1359.29 | 550.56 | 714.45 |
|  | Minimum | 1042.65 | 696.96 | 277.32 | 3.94 |
|  | Standard deviation | 228.33 | 135.71 | 56.34 | 148.95 |
|  | Coefficient of variation | 0.1631 | 0.1495 | 0.1532 | 1.0333 |

Table S3: Statistical values by indicators for the three clusters (SSP3-7.0, 2021–2050).

| Classification | Statistical value | Average annual rainfall(mm) | Average rainfall during the rainy season(mm) | Average monthly maximum rainfall(mm) | Elevation(m) |
| --- | --- | --- | --- | --- | --- |
| Cluster 1  (n = 27) | Average | **1571.67** | **1010.49** | **437.51** | **82.72** |
|  | Maximum | 1991.79 | 1313.12 | 567.43 | 478.65 |
|  | Minimum | 1331.95 | 850.11 | 340.68 | 6.67 |
|  | Standard deviation | 170.50 | 114.50 | 52.12 | 96.03 |
|  | Coefficient of variation | 0.1084 | 0.1133 | 0.1191 | 1.1609 |
| Cluster 2  (n = 53) | Average | **1165.21** | **776.35** | **330.88** | **89.06** |
|  | Maximum | 1454.93 | 991.92 | 422.43 | 316.39 |
|  | Minimum | 984.92 | 664.00 | 289.78 | 3.94 |
|  | Standard deviation | 110.69 | 69.99 | 31.28 | 67.79 |
|  | Coefficient of variation | 0.0950 | 0.0901 | 0.0945 | 0.7612 |
| Cluster 3  (n = 21) | Average | **1389.45** | **941.99** | **400.00** | **362.14** |
|  | Maximum | 1606.31 | 1058.65 | 456.21 | 714.45 |
|  | Minimum | 1221.01 | 835.01 | 354.92 | 173.01 |
|  | Standard deviation | 101.05 | 62.61 | 26.72 | 152.36 |
|  | Coefficient of variation | 0.0727 | 0.0664 | 0.0668 | 0.4207 |
| All  (n = 101) | Average | **1320.49** | **873.38** | **373.75** | **144.14** |
|  | Maximum | 1991.79 | 1313.12 | 567.43 | 714.45 |
|  | Minimum | 984.92 | 664 | 289.78 | 3.94 |
|  | Standard deviation | 216.4 | 133.55 | 59.85 | 148.95 |
|  | Coefficient of variation | 0.1638 | 0.1529 | 0.1601 | 1.0333 |

Table S4: Statistical values by indicators for the three clusters (SSP3-7.0, 2051–2100).

| Classification | Statistical value | Average annual rainfall(mm) | Average rainfall during the rainy season(mm) | Average monthly maximum rainfall(mm) | Elevation(m) |
| --- | --- | --- | --- | --- | --- |
| Cluster 1  (n = 27) | Average | **1687.79** | **1044.19** | **427.76** | **82.72** |
|  | Maximum | 2128.24 | 1346.1 | 546.99 | 478.65 |
|  | Minimum | 1427.99 | 907.64 | 363.18 | 6.67 |
|  | Standard deviation | 181.14 | 111.08 | 44.28 | 96.03 |
|  | Coefficient of variation | 0.1073 | 0.1063 | 0.1035 | 1.1609 |
| Cluster 2  (n = 53) | Average | **1240.33** | **794.95** | **324.21** | **89.06** |
|  | Maximum | 1547.88 | 984.89 | 401.44 | 316.39 |
|  | Minimum | 1040.13 | 673.06 | 277.08 | 3.94 |
|  | Standard deviation | 121.67 | 71.76 | 28.9 | 67.79 |
|  | Coefficient of variation | 0.0981 | 0.0902 | 0.0891 | 0.7612 |
| Cluster 3  (n = 21) | Average | **1470.28** | **954.93** | **382.43** | **362.14** |
|  | Maximum | 1720.84 | 1100.59 | 439.77 | 714.45 |
|  | Minimum | 1294.33 | 846.06 | 329.69 | 173.01 |
|  | Standard deviation | 106.66 | 66.69 | 26.85 | 152.36 |
|  | Coefficient of variation | 0.0725 | 0.0698 | 0.0702 | 0.4207 |
| All  (n = 101) | Average | **1407.76** | **894.84** | **364** | **144.14** |
|  | Maximum | 2128.24 | 1346.1 | 546.99 | 714.45 |
|  | Minimum | 1040.13 | 673.06 | 277.08 | 3.94 |
|  | Standard deviation | 235.35 | 137.38 | 55.68 | 148.95 |
|  | Coefficient of variation | 0.1671 | 0.1535 | 0.1529 | 1.0333 |

Table S5: Homogeneity test and trend analysis of Hapcheon Dam inflow (2021–2050).

| Division | SSP2-4.5 | | | SSP3-7.0 | | |
| --- | --- | --- | --- | --- | --- | --- |
|  | Dam inflow trend  (Homogeneity test) | Rainfall trends at nearby stations | Number of nearby stations showing trends | Dam inflow trend  (Homogeneity test) | Rainfall trends at nearby stations | Number of nearby stations showing trends |
| January | −  (−) | − | − | −  (Pass) |  | − |
| February | −  (Pass) | − | − | −  (Pass) |  | − |
| March | −  (Pass) | − | − | −  (Pass) |  | − |
| April | −  (−) | − | − | −  (Pass) |  | − |
| May | −  (−) | − | − | −  (Pass) |  | − |
| June | −  (Pass) | − | − | −  (Pass) |  | − |
| July | −  (Pass) | Decrease (↓) | 3 | −  (Pass) | Increase (↑) | 3 |
| August | −  (Pass) | − | − | −  (Pass) |  | − |
| September | −  (Pass) | − | − | −  (Pass) |  | − |
| October | −  (Pass) | − | − | −  (Pass) |  | − |
| November | −  (Pass) | − | − | −  (Pass) |  | − |
| December | −  (Pass) | − | − | −  (Pass) |  | − |
| Spring | −  (−) | − | − | −  (Pass) |  | − |
| Summer | −  (Pass) | − | − | −  (Pass) |  | − |
| Fall | −  (Pass) | − | − | −  (Pass) |  | − |
| Winter | −  (Pass) | − | − | −  (Pass) |  | − |
| Year | −  (Pass) | − | − | −  (Pass) | Increase (↑) | 1 |

Table S6: Homogeneity test and trend analysis of Hapcheon Dam inflow (2051–2100).

| Division | SSP2-4.5 | | | SSP3-7.0 | | |
| --- | --- | --- | --- | --- | --- | --- |
|  | Dam inflow trend  (Homogeneity test) | Rainfall trends at nearby stations | Number of nearby stations showing trends | Dam inflow trend  (Homogeneity test) | Rainfall trends at nearby stations | Number of nearby stations showing trends |
| January | −  (Pass) | − | − | −  (Pass) | − | − |
| February | −  (Pass) | − | − | −  (−) | − | − |
| March | −  (Pass) | − | − | −  (Pass) | − | − |
| April | −  (Pass) | Increase (↑) | 2 | −  (Pass) | − | − |
| May | −  (Pass) | − | − | −  (Pass) | − | − |
| June | −  (−) | − | − | −  (Pass) | − | − |
| July | −  (Pass) | − | − | −  (Pass) | − | − |
| August | −  (Pass) | − | − | −  (Pass) | Increase (↑) | 4 |
| September | −  (Pass) | − | − | −  (Pass) | − | − |
| October | −  (Pass) | − | − | −  (Pass) | − | − |
| November | Increase (↑)  (Pass) | − | − | −  (Pass) | − | − |
| December | −  (−) | − | − | −  (Pass) | Decrease (↓) | 10 |
| Spring | −  (Pass) | Increase (↑) | 2 | −  (Pass) | − | − |
| Summer | −  (Pass) | − | − | −  (Pass) | − | − |
| Fall | −  (Pass) | − | − | −  (Pass) | − | − |
| Winter | −  (Pass) | − | − | −  (−) | − | − |
| Year | −  (Pass) | − | − | −  (Pass) | − | − |


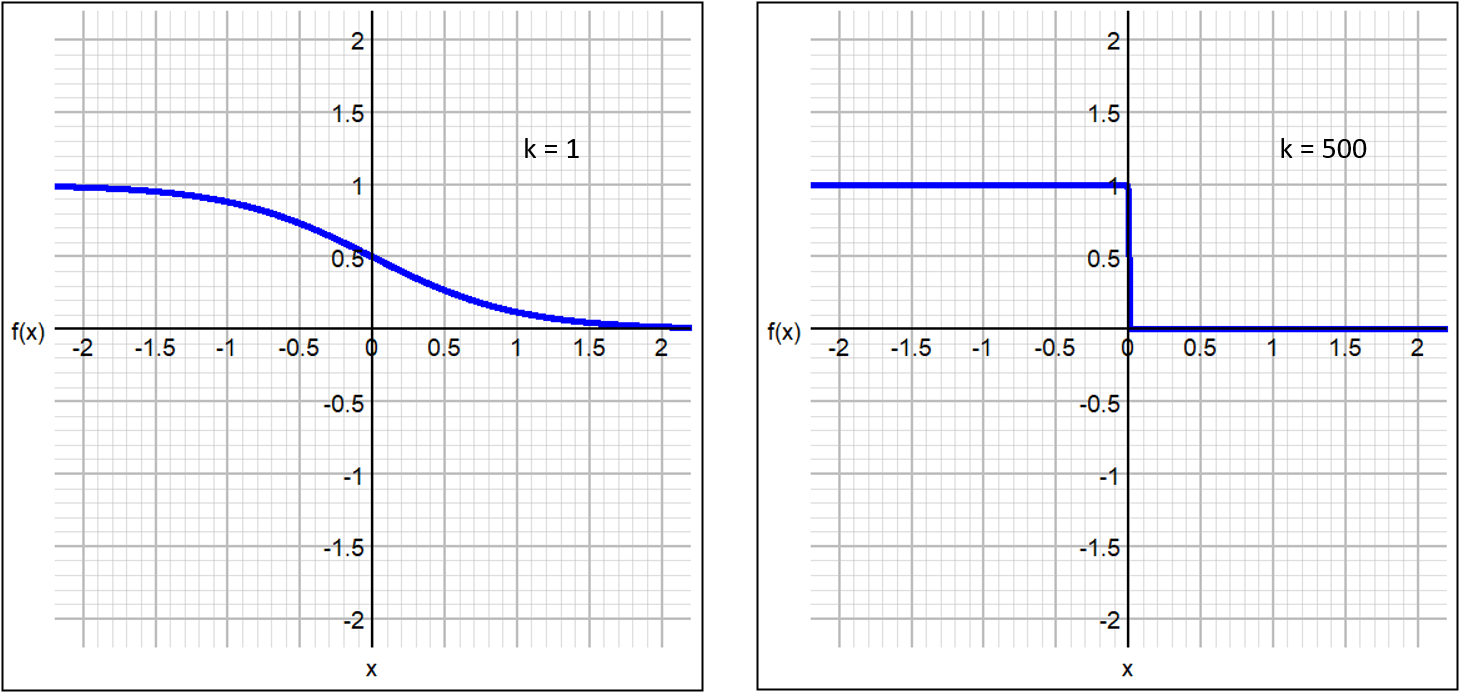


Fig. S1: Changes in the $\boldsymbol{f}\left( \boldsymbol{x} \right)$ graph according to $\boldsymbol{k}$.


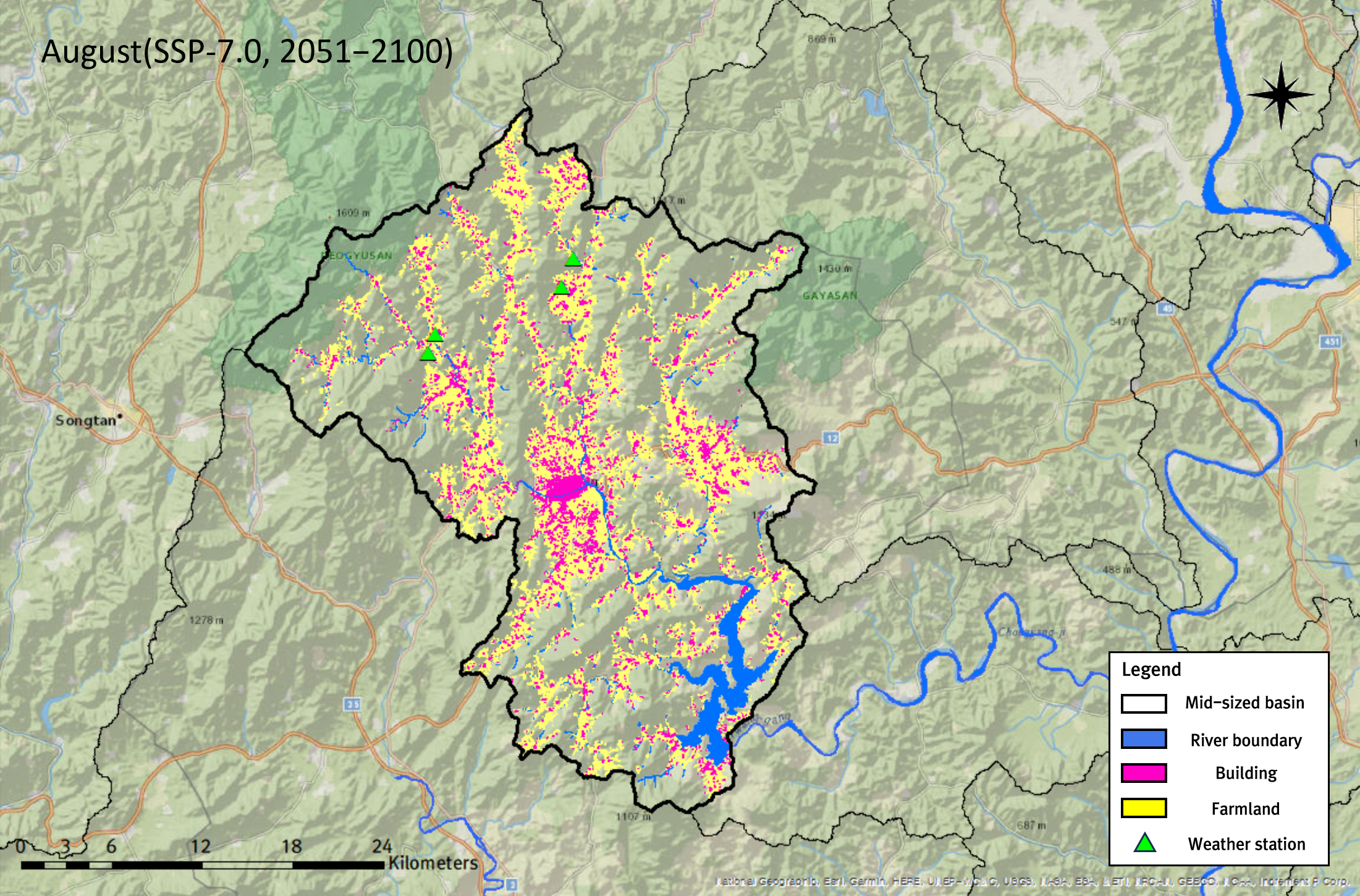


Fig. S2: Distribution of the 4 weather stations that show an increasing trend in the Hapcheon Dam Basin. The map was created using ArcGIS Pro 3.6.1 (https://www.esri.com/en-us/arcgis/products/arcgis-pro/overview) with the National Geographic World Map basemap.


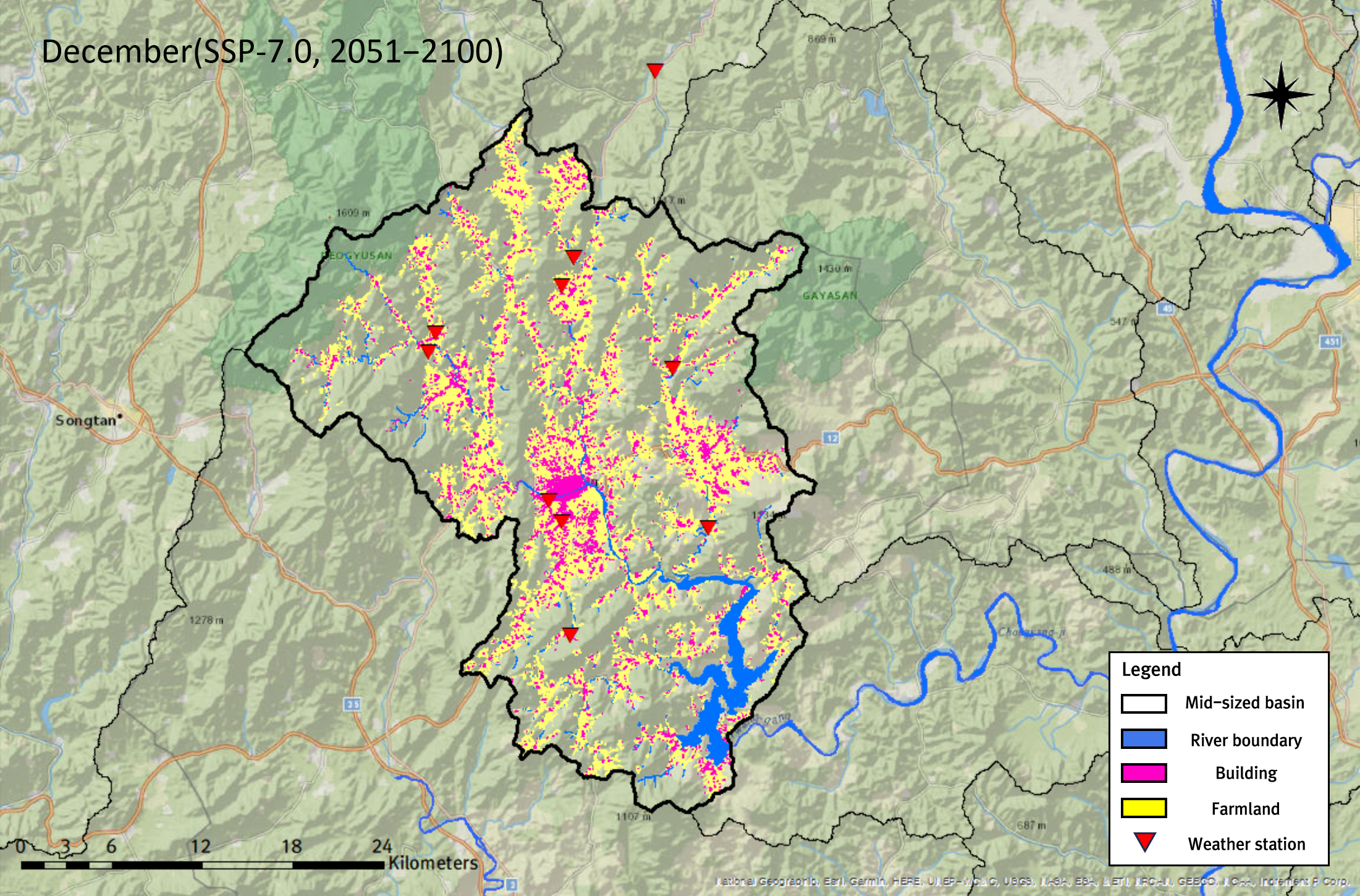


Fig. S3: Distribution of the 10 weather stations that show a decreasing trend in the Hapcheon Dam Basin. The map was created using ArcGIS Pro 3.6.1 (https://www.esri.com/en-us/arcgis/products/arcgis-pro/overview) with the National Geographic World Map basemap.


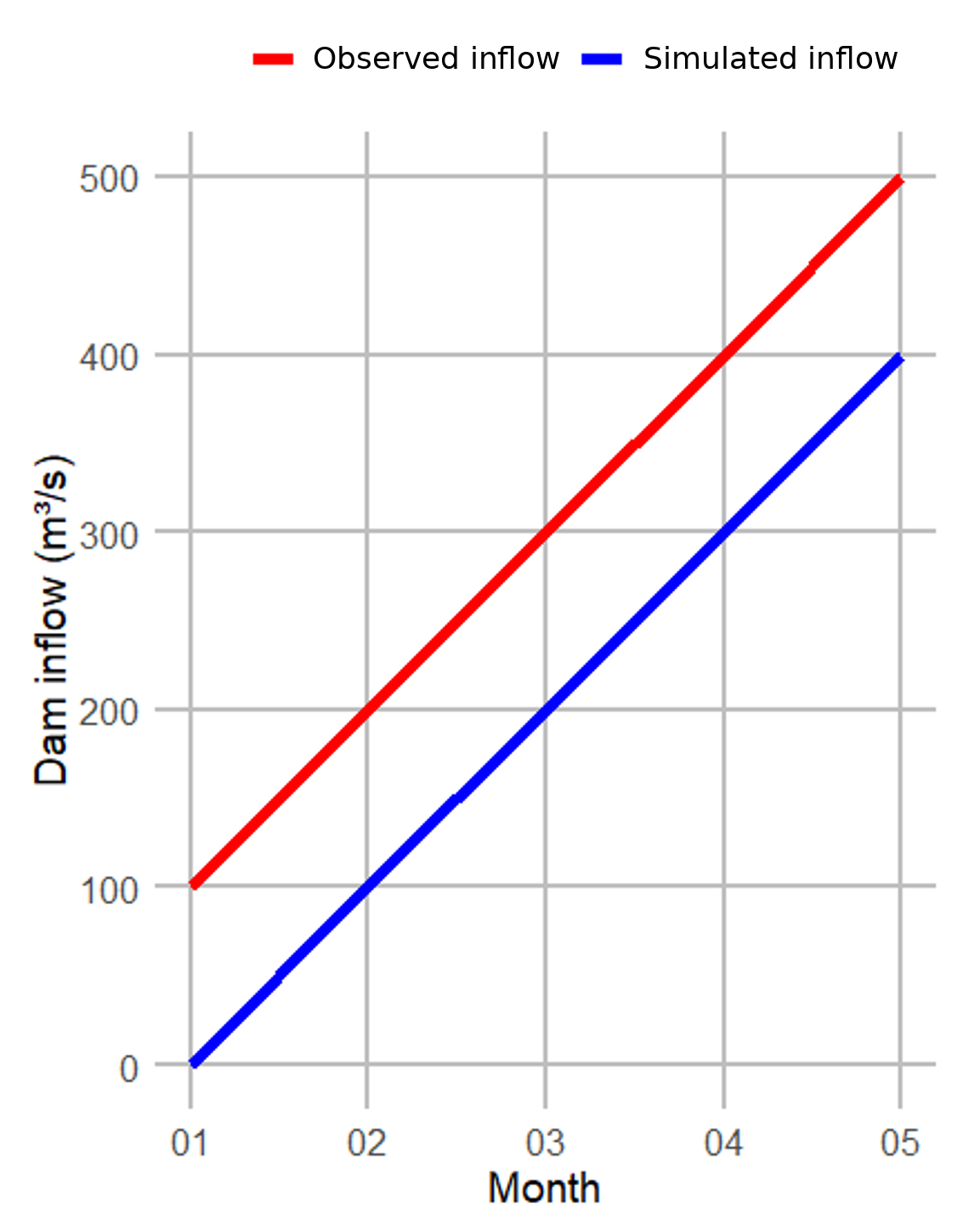


Fig. S4: Example of observed and simulated dam inflows (January–May).
